# Effect of an Appearance-Based vs. a Health-Based Sun-Protective Intervention on French Summer Tourists' Behaviors in a Cluster Randomized Crossover Trial: The PRISME Protocol

**DOI:** 10.3389/fpubh.2020.569857

**Published:** 2020-11-05

**Authors:** Cécile Durand, Olivier Catelinois, Apolline Bord, Jean-Baptiste Richard, Marie-Laure Bidondo, Colette Ménard, Florence Cousson-Gélie, Emmanuel Mahé, Damien Mouly, Cyrille Delpierre

**Affiliations:** ^1^Santé Publique France (SpF), Regions Division, Occitanie, Toulouse, France; ^2^UMR1027, Université de Toulouse, UPS, Inserm, Toulouse, France; ^3^Institut du Cancer de Montpellier (ICM), Prevention Department Epidaure, Montpellier, France; ^4^Santé Publique France (SpF), Support, Processing and Data Analysis Division, Saint-Maurice, France; ^5^Santé Publique France (SpF), Health Prevention and Promotion Division, Saint-Maurice, France; ^6^Université Paul Valéry Montpellier 3, Université Montpellier, EPSYLON EA 4556, Montpellier, France; ^7^Hospital center of Argenteuil—Dermatology Department, Argenteuil, France

**Keywords:** ultraviolet exposure, sun protection, prevention, behaviors, cluster randomized crossover trial, tourists, appearance-based interventions, ultraviolet photographs

## Abstract

**Background:** Sun exposure has short- and long-term adverse effects on eyes, skin, and the immune system. The most serious effect, melanoma, is largely attributable to natural ultraviolet radiation. Its prevalence is steadily increasing in fair-skinned populations in most European countries. Despite annual prevention campaigns, the French population continues to be overexposed to the sun and under-protected. Social and psychosocial characteristics may play an important role in sun protection determinants. Overexposure is partially motivated by a desire to tan oneself for aesthetic reasons. During summer, intense exposure constitutes a major risk factor for melanoma, making tourists a particularly high-risk population. Literature reviews concluded that appearance-based interventions highlighting the aesthetic effects of sun exposure on skin photoaging showed promise in terms of improving sun-exposure and sun-protection behaviors, especially among younger people, but that more rigorous studies were needed. In this context, we implemented the PRISME study to:

- identify the determinants, in particular social and psychosocial, of sun-protection of French summer tourists visiting the Mediterranean coastline;

- design two prevention interventions grounded in psychosocial theories;

- compare the impact of both interventions on tourists' sun-protection behaviors, and identify the determinants influencing this impact.

This paper presents the methodology of the PRISME study.

**Methods:** During summer 2019, we conducted a cluster randomized crossover trial to compare two prevention interventions, one based on health-related messages (health effects information, phototype calculation), the other on appearance-related messages (photoaging information, ultraviolet photography), among French tourists aged 12–55 years old in coastline campsites in the French region of Occitanie. Both interventions were anchored in the theory of planned behavior and in the transtheoretical model. The interventions' impact was measured using face-to-face questionnaires and skin color measurements both immediately before and 4 days after the interventions. A second follow-up, using an online questionnaire, will be conducted in September 2020 to measure the longer-term effects of both interventions.

**Discussion:** Despite certain study limitations, PRISME take into consideration several known methodological gaps. The study's results will enable to evaluate the efficacy of the promising appearance-based approach in France, and to identify vulnerable sub-populations and mechanisms to improve sun-protection behaviors of French tourists.

## Introduction

### Sun Exposure: Short- and Long-Term Effects

Although sun exposure has beneficial health effects, including vitamin D synthesis and well-being, it is also associated with short- and long-term adverse effects on the eyes, skin, and immune system (1, 2).

Short-term effects on the eyes include photokeratitis and photoconjunctivitis, while long-term effects include age-related macular degeneration, eye cancer and cataracts. The World Health Organization (WHO) estimates that 20% of cataracts may be due to overexposure to ultraviolet rays (2).

With respect to skin, early photoaging is characterized by a loss of elasticity, roughness, wrinkles, telangiectasias, sunspots, and irregular pigmentation, mainly on the parts of the body most exposed, like the face, the back of the hands and forearms. Long-term adverse effects include carcinomas and melanomas. These two cancer types account for 1/3 of all cancers and, although the data are imprecise for carcinomas, the total number of new cases of skin cancer diagnosed worldwide each year is estimated at between 2 and 3 million^1^, and between 101,000 and 160,000 in France in 2012 (3).

The most serious skin cancer, melanoma, is unequally distributed around the world. Incidence is 10 times higher in developed countries, especially Australia–New Zealand, North America, and Europe (4). It has steadily increased among fair-skinned populations over the last 50 years, especially in most European countries, although the incidence in some countries has recently stabilized (4). In France in 2018, the number of new annual cases is estimated at 15,500, while the estimated number of related deaths was 1,975 in 2018 (5). Melanoma is one of the most common cancers among young adults, with incidence increasing from the age of 30 onwards in both sexes (5). In France, 83% of melanomas are attributable to natural ultraviolet radiation (6), an exposure type which concerns the entire population. Skin cancers, particularly melanomas, are the result of inappropriate and repeated sun exposure, especially before the age of 15 (1). There is also an increased risk after intermittent and intense sun exposure such as that experienced during summer holidays (1). This makes tourists a particularly vulnerable population.

### Summer Sun Overexposure for Aesthetic Reasons

Overexposure to the sun during the summer in the French tourist population is partially driven by their desire to tan for aesthetic reasons (7, 8). Until the end of the 19th century, white skin was considered a sign of higher social class, tanned skin being associated with people with lower socioeconomic status who worked in the fields. With the industrial revolution, the post-war liberation, and the beginning of paid holidays in 1936, the French population gradually increased their exposure to the sun, to the point where tanning became a symbol of leisure and financial comfort (8, 9). This social norm evolved until the 1990s with suntans continuing to be featured in the women's press. Today, excessive tanning is less fashionable, but a moderate tan is still a positive social norm associated with beauty, health, and well-being (7).

### French Sun Protection Still Insufficient

Despite national public health campaigns every spring since 1996 focusing on the dangers of overexposure to the sun and ways to protect against it, the French continue to overexpose themselves. They do not systematically use preventive skin-protection, especially adolescents and young adults (3). Some sun protection resources are preferred (sunscreen, glasses) at the expense of others which are more effective (shade, t-shirt, hat). Many false beliefs persist about sun protection, and some are becoming even more widespread. Furthermore, knowledge and sun protection behaviors are dependent on an individual's social and psychosocial characteristics (3, 10). These characteristics may play an important role in sun exposure and protection determinants (3, 10). However, French data on this subject are still scarce.

### Promising Appearance-Based Interventions in the Young Tourist Population

An analysis of the existing literature on sun-prevention interventions reveals different approaches including: (1) individual-directed strategies, like interventions based on health education messages or on the negative aesthetic effects of ultraviolet exposure, (2) interventions to improve the environment like creating shaded areas, or sun-safety policies like scheduling of outdoor activities to avoid peak ultraviolet hours, (3) media campaigns and (4) community-wide multicomponent programs (11, 12). The first two approaches can be implemented in various settings and populations like schools, families, occupational settings, healthcare system, and recreational and tourism contexts. In the latter two settings, sun-prevention interventions seem to be effective at increasing adult and children sun-protection behaviors (11, 12), but the results and quality of related studies to date are heterogeneous, suggesting possible bias (13).

Given the positive societal perception of tanning detailed above, the literature suggests that appearance-based interventions highlighting the negative aesthetic effects of ultraviolet exposure may be more effective than other intervention approaches (13) at increasing sun-protection intention and behaviors, and sometimes at reducing exposure (14–16). Furthermore, the positive effects of this type of intervention would appear to be stronger when both photoaging information and ultraviolet photographs are used (16). This greater efficacy of appearance-based interventions is all the more true for tourists, a population particularly fond of tanning, and for young adults and adolescents who see health risks as something to be concerned about only in the distant future (17, 18). However, a recent review of the literature (16) concluded that in order to properly evaluate appearance-based interventions, further studies were needed with theoretically constructed interventions, a sufficient sample based on power calculation, a longer follow-up period, and with populations of various ages, genders, and ethnicities. Moreover, as related studies have mostly been carried out in Anglo-Saxon countries, one would need to evaluate the transferability of this type of approach to the French context where social norms and the environmental context are different. It should be noted that sun-protection interventions are still rare in France, despite the country's long coastline and large amount of sunshine in many areas.

### French Mediterranean Coastline: A Prominent Tourist Area With Strong Ultraviolet Radiation

The French Mediterranean coastline has high levels of ultraviolet radiation and is a prominent summer destination for tourists. The 200 km stretch of coastline in the Occitanie region has 21 million stays each summer. More than 75% of these are in campsites and 75% of campsite tourists are French nationals^2^. Campsites on the Occitanie coastline therefore represent a particularly relevant context to analyze sun exposure and protection behaviors of French tourists.

Accordingly, the PRISME (PRevention and Impact of Sun exposure on the French MEditerranean coast) study was implemented in 2019, with the aim of improving sun protection in French summer tourists.

The specific objectives were:

- to identify the determinants, in particular the social and psychosocial determinants, of sun-protection of French summer tourists on the Mediterranean coastline;
- to design two interventions—one health-based, the other appearance-based—both grounded in psychosocial theories;
- to compare the impact of the interventions on French tourists' sun-protection behaviors and intentions, and to identify the social and psychosocial determinants influencing the impact of both interventions.

PRISME's underlying hypothesis was that an appearance-based intervention would be more effective in changing sun protection behaviors and intentions than a health-based intervention, especially among younger tourists. It was also hypothesized that the impact of these two types of intervention may differ according to individual profiles, in particular social and psychosocial characteristics.

The objective of this paper is to present the methodology used in the PRISME study.

## Methods and Analysis

### Design: Cluster Randomized Crossover Trial

This intervention study design consisted of a cluster randomized crossover trial. Participants could belong to three different interventions groups:

- Group 0: no intervention (control group)
- Group 1: health-based intervention (intervention 1)
- Group 2: appearance-based intervention (intervention 2).

### Study Setting: Enrollment in Coastal Campsites During the Summer

Inclusion at time T0 and the first follow-up T1 (T0 + 3–4 days) took place in eight campsites along the Occitanie coastline from July 7 to August 30, 2019. We randomly selected campsites in six strata based on the official campsite classification (0–2 stars, 3 stars, 4–5 stars) and location (northern and southern zone of the region) (Table 1). By selecting a variety of campsites in different strata, we were able to include populations from various socioeconomic groups exposed to potentially different climatic conditions during the study period. A second follow-up T2 will be performed by email at the end of the summer 2020 to measure longer-term changes in sun-protection behaviors, as recommended in the literature (16, 19).

**Table 1:** Allocation of intervention groups by campsites and week—PRISME.

**Zone**	**Classification**	**Pitches**	**Strata number**	**Campsite**	**W28**	**W29**	**W30**	**W31**	**W32**	**W33**	**W34**	**W35**
Northern zone	⋆⋆	219	2	Campsite 1	0	0	2	2	W	1	1	W
	⋆⋆⋆⋆	168	6	Campsite 2	1	W	0	0	2	2	2^**^	2^*^
	⋆⋆⋆	718	4	Campsite 3	W	1	1	W	0	0	2	2
	⋆⋆⋆⋆	450	6	Campsite 4	2	2	W	1	1	W	0	0
Southern zone	⋆⋆⋆⋆	268	5	Campsite 5	0	0	2	2	W	1	1	1^**^
	⋆⋆⋆⋆	590	5	Campsite 6	1	W	0	0	2	2	2^**^	1
	⋆⋆⋆	139	3	Campsite 7	W	1	1	W	0	0	2	2
	⋆⋆	215	1	Campsite 8	2	2	W	1	1	W	0	0
*Number of participants included at T0*					*68*	*97*	*175*	*220*	*234*	*209*	*210*	*142*

W, wash-out week where no data were collected.

* Protocol adaptation: initially Group 1 becoming Group 2.

** *Protocol adaptation: initially wash-out week becoming data collection week*.

### Participants: French Tourists 12–55 Years Old

The study population comprised French tourists from 12 to 55 years old, staying in one of the selected campsites. Additional exclusion criteria were as follows: not a French speaker, living abroad, health problems totally forbidding sun exposure, departure before 4 days after inclusion (which would have prevented participation in T1), and for minors, staying in the campsite without a legal guardian.

### Two Theoretically Constructed Interventions Based on Health Messages and Appearance

**Figure 1:**
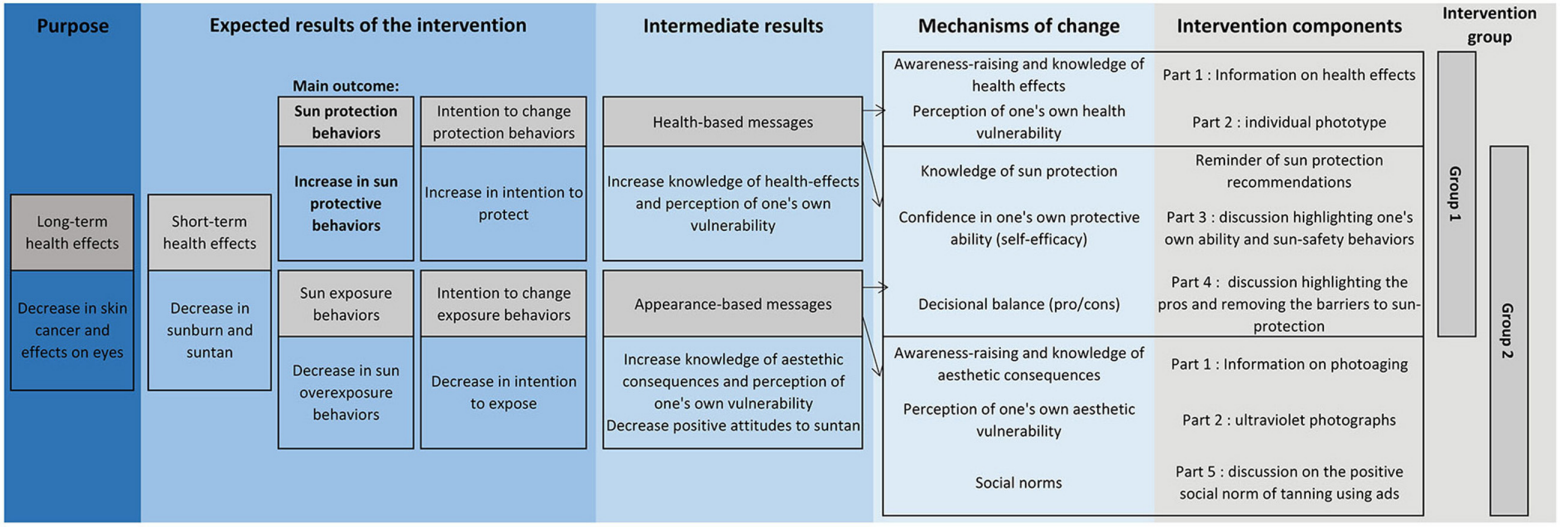
Intervention logic model—PRISME.

In line with the literature, we developed an intervention logic model (Figure 1) and constructed two interventions based on similar mechanisms but communicating different messages. The first intervention used health messages (intervention 1) while the second used messages focusing on the consequences of sun exposure on appearance (intervention 2).

The interventions were constructed from several mechanisms and both grounded in two theories of behavioral change as follows (20): (a) modification of attitudes, social norms and perceived control (self-efficacy) influencing behavior according to the theory of planned behavior (21), and (b) consciousness raising, dramatic relief, self-efficacy and decisional balance contributing to the process of change according to the transtheoretical model (22). Implementing interventions based on psychosocial theories is one way to identify which intervention mechanisms are associated with targeted sun-protective behaviors, thus enabling the selection of effective intervention techniques.

Different intervention booklets were used for intervention 1 and 2 (Supplementary Materials 1, 2).

Both interventions consisted of different parts as follows: providing information about the consequences of sun exposure using pictures in order to increase knowledge (part a), a self-evaluation of one's own vulnerability to the sun in order to raise awareness (part b), a reminder of the different protection recommendations, a discussion on the participant's current sun protection behaviors in order to increase his self-confidence regarding protection (i.e., self-efficacy) (part c), and finally, a discussion on the pros and cons of using means of protection non-currently used by the participant (decisional balance) (part d). In intervention 2, we also added an additional part (part e) which consisted in discussing the impact of the social norm of tanning using ads and social media.

For parts a and b, the tools and messages used were different between the two interventions:

- In intervention 1, part a contained information about health risks (sunburn, eyes problems, cancer), while negative aesthetic effects and photoaging were the focus in intervention 2.
- In intervention 1, part b evaluated individual sensitivity by calculating phototype using a quiz. Instead, in intervention 2, individual sensitivity was approached by face and profile ultraviolet photographs to visualize skin damage (spots) not visible to the naked eye. These photographs were printed and given to the participant (23). Four Canon EOS 200D™ reflex cameras were used. They were converted into full-spectrum by a specialized company, with Nikon Nikkor™ AF 50F/1.8D camera lenses and Kolari vision™ UV-bandpass filters (320–400 nm).

In addition to these different parts, additional elements were available in the intervention booklets as follows: suggestions for alternative activities between noon and 4 pm to avoid exposure to the sun, raising parent awareness of their role in protecting young children, information on sun radiation and the ultraviolet index, quiz, and games.

Both interventions emphasized a hierarchy in the sun-protection behaviors to implement. The most important behavior is to reduce exposure, by seeking shade and avoiding sun between noon and 4 pm. The second is to put on a t-shirt, hat, and sunglasses while in the sun outside these hours, and finally, as a last resort, the use of sunscreen minimum SPF30 (Sun Protection Factor) on exposed body parts.

The interventions were implemented face-to-face in the study's camping sites by 10 specialized prevention workers (in the fields of health, psychology, social, and leisure) previously trained on sun prevention. Prevention workers involved in intervention 2 were also previously trained to take UV photographs by a specialist photographer^3^. Furthermore, a supervisor specialized in sun prevention was available by phone or on site throughout the study period to provide assistance and supervise the prevention workers. Problems linked to adherence to the intervention protocol (e.g., participant's refusal to have ultraviolet photographs taken, incomplete intervention due to lack of time of the participant, etc.) were noted on a prevention summary record (Supplementary Material 3). At the end of each intervention, participants were given a gift (tote bag and a pen).

**Figure 2:**
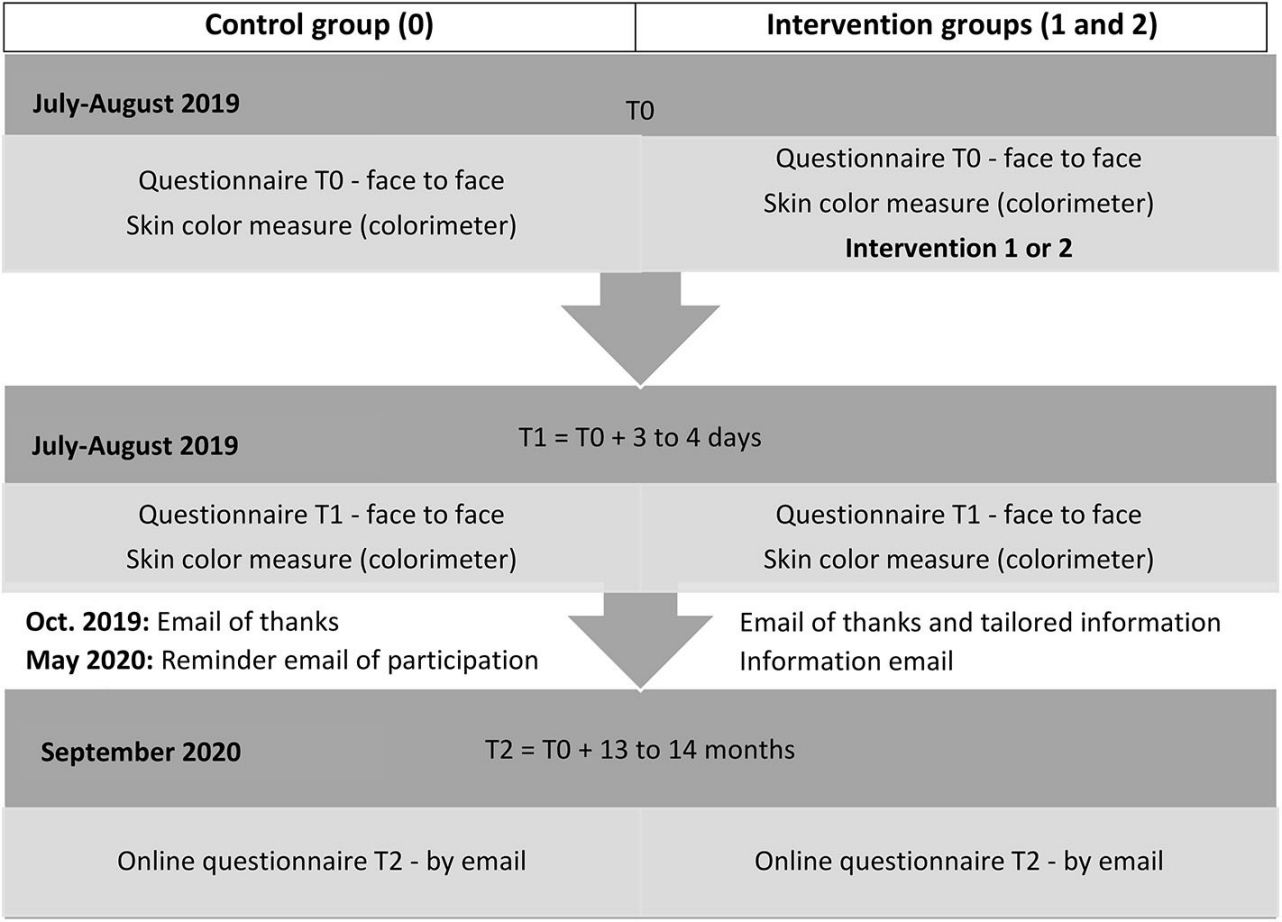
Study timeline—PRISME.

In October 2019, an email was sent to all participants (Figure 2). For Group 1, the email contained information on the health risks of sun exposure and detailed the participant's phototype, as determined during the intervention at T0. Individually tailored sun-safety messages were also included according to this phototype. For Group 2, the email provided information on photoaging and the individual ultraviolet photographs taken during the intervention. Sun-safety messages were also included.

In May 2020, before the summer season, a new email will be sent to the participants including the same information but insisting on the importance of adopting sun-safety behaviors for the upcoming summer season (Figure 2).

The creation of intervention materials (e.g., the booklet, tote bag, etc.), together with the recruitment, training and supervision of prevention workers was undertaken by the Prevention Department of the Montpellier Cancer Institute (Epidaure), which is specialized in prevention and health education^4^.

### Outcomes

There are a large number of possible outcomes for sun-protection interventions (11, 19), and the impact can be measured using specific instruments, external observers and patient-reported information. The most common outcomes are: protective behaviors (self-reported or observed *in situ*), exposure behaviors (self-reported or measured using dosimeters for example), intention to change protection and/or exposure behaviors (self-reported), short-term health effects (sunburn, suntan, nevus) (self-reported or measured), and long-term health effects (self-reported or measured).

The impact of each of the two interventions in the PRISME study is evaluated by self-reported indicators using a standardized questionnaire and by colorimeter (described below).

The study's outcomes are as follows:

- The main outcome is a composite indicator of various self-reported protective behaviors. More specifically, a person is considered fully protected if his self-reported sun-protection behaviors reflect current prevention recommendations: staying (systematically) in the shade, or avoiding (systematically or often) sun-exposure between noon and 4 pm using (systematically or often) all the recommended sun-protection resources (t-shirt, hat, sunglasses) and applying (systematically or often) sunscreen on exposed body parts every 2 h.
- The secondary outcomes are (1) self-reported exposure behaviors, for example the number of hours in the sun, especially in high-risk contexts (between noon and 4 pm, at the beach or swimming pool, sunbathing), (2) a self-reported short-term health effect indicator with sunburn frequency during the holidays, and (3) two self-reported indicators of intention to change behaviors (as per the transtheoretical model) (17, 24), specifically an intention to improve sun protection and an intention to stop sunbathing in the future.
- Moreover, skin color was measured using a colorimeter at T0 and T1, to quantify short-term suntan, and possibly sunburn, during the campsite vacation period. Skin color constitutes a non-declarative indicator that creates less social desirability bias than self-reported behaviors indicators (19, 25). The skin color indicators to be analyzed will are: (i) the individual typology angle (ITA°) which represents the general skin color, (ii) the L^*^ value of the CIE L^*^a^*^b^*^ color space, and the melanin index which both represent the level of tanning, and (iii) the a^*^ value of the CIE L^*^a^*^b^*^ color space and erythema index which both represent the level of erythema (26).

### Participant Timeline: Three Data Collection Timepoints

#### Baseline T0

Participants answered a 20-min standardized questionnaire, administered face to face by an interviewer, at the beginning of the week (Sunday/Monday/Tuesday) (T0). A double measurement of skin color at 4 measurement points (shoulder, inner arm, cheekbone, nose) was also performed by the interviewer in the same moment. In groups 1 and 2, the interviewer worked together with a prevention worker who subsequently delivered the relevant intervention (i.e., health-based or appearance-based) in approximately 20–25 min.

#### T1 Follow-Up

Three to four days after inclusion (Thursday/Friday), the interviewer returned alone to the participant's pitch at a scheduled time in order for the participant to complete a second face-to-face questionnaire, which lasted between 10 and 15 min. The interviewer also measured skin color again using the same procedure as in T0.

#### T2 Follow-Up

An intervention email was sent in October 2019 and will be followed by another in May 2020. Subsequently, we will send a third questionnaire by email in September 2020. This self-administered questionnaire will permit us to measure the longer-term impact of both interventions (Figure 2).

### Questionnaires and Colorimeter

The T0 questionnaire (Supplementary Material 4) included items collecting sociodemographic and physical data, email, as well as questions about knowledge of and attitudes to sun-protection, current exposure and protection behaviors and intention to change. It also included questions for parents with children under 12 years of age about the sun protection of their youngest child, in order to indirectly assess children's sun protection through their parents' behaviors.

Questions focusing on knowledge, attitudes, current protection, and exposure behaviors were taken (either directly or adapted) from the questionnaire used in the annual French Health Barometer survey (3) and from international literature (27–30). Intention to change questions were adapted from existing international literature using the stages of change of the transtheoretical model (17, 24, 27). Phototype was recorded according to the Fitzpatrick classification (31), adapted as per recent literature (32, 33).

The T1 questionnaire (Supplementary Material 5) included questions about knowledge, attitudes, intention to change, behaviors during the campsite vacation in 2019 and short-term health effects related to the sun occurring in the participant and their youngest child under 12.

The T2 questionnaire in September 2020 will include the same questions as in T1 but will concern behaviors and health effects observed during the summer of 2020.

The colorimeters used at T0 and T1 were SkinColorCatch™ by Delfin Technologies Ltd. They measured the skin color in six chromatic values: CIE L^*^a^*^b^*^, RGB, L^*^c^*^h^*^ color space, melanin index, erythema index, and ITA°^5^ (26, 34).

Before the beginning of the study, a pilot study was conducted for 1 week in April 2019 in two of the 8 campsites with 18 participants in order to test the questionnaire, the interventions, and the measurement of skin color. Some participants considered the pilot questionnaire to be too long. Accordingly, we deleted and modified certain questions and adapted the procedure for collecting and transmitting skin color data.

### Calculating Required Sample Size

We calculated a required sample size *a priori* (35) using the following hypothesis: an eligibility rate of 60% (estimated with input from campsite managers), a participation rate of 60% at inclusion, a retention rate of 40% at T2 follow-up (24), a cluster effect (RHO) of 0.05, a period effect (ETA) of 0.04, a power of 80%, and an alpha risk of 5%. To observe a difference of 10 points for the main outcome, and therefore to increase from 30% [(3) additional unpublished data] to 40% the frequency of fully protected participants (defined above), it was necessary to enroll 8 campsites and obtain a final sample of 328 campsite pitches for each intervention group (Groups 0, 1, and 2), that is to say 984 campsite pitches in total.

### Intervention Allocation: Alternation Between Intervention Groups by Week and by Campsite

To limit inter-group contamination by communication between participating families, instead of individual allocation, we allocated a single intervention group to all participants in a particular campsite for a given week.

In addition, we chose a crossover design whereby the allocation of intervention groups was alternated for each campsite on a weekly basis during the 8 weeks of the study, rather than a parallel design in which the same group would have been allocated to a given campsite throughout the summer. The crossover design takes greater account of the large disparity between campsites in terms of populations and urban infrastructure, two factors which influence sun exposure. We also included washout weeks, especially between control groups and intervention groups, to limit possible inter-group contamination by tourists staying for several weeks (Table 1).

Participants, interviewers, and prevention workers knew the allocation group they were involved in. However, participants had very little information about other intervention groups and prevention workers were trained for and implemented only one intervention.

### Recruitment and Randomization of Campsite Pitches and Participants

The campsite pitches were randomized each week in each campsite using two combined methods: randomly choosing pitches currently occupied from the campsite's booking lists, and randomly choosing pitches from all existing pitches on the campsite in order to include people who arrived without a reservation. In order to reach the required participation numbers, the interviewers had main and reserve lists of randomly selected campsite pitches. They systematically used the main list, but could use from 1 to 7 additional reserve lists (of 5 pitches each) as soon as 5 campsite pitches which they approached were definitively unusable (because of repeated absence, refusal, and ineligibility). Any reserve list started had to be fully exploited. A campsite pitch was considered absent after three contactless visits at different times on different days.

Once contact was established at a campsite pitch and agreement to participate obtained by the occupants, the interviewers checked for eligibility. The interviewer's tablet, used for the survey, randomly drew one adult (18–55 years old) and, if present, a teenager (12–17 years old) from the persons eligible. Selected individuals still had the possibility to refuse participation at this point.

### Data Management and Confidentiality

For data collection until T1, we collaborated with the Ipsos Institute, a French study and market company with ISO 9001 version 2008 and ISO 20252 version 2012 quality certification. Professional interviewers previously trained for this survey collected data in real time on secure tablets using Ifield software. Two supervisors were present in the campsites to monitor and manage the interviewers' work. Data was transmitted and stored directly on Ipsos' secure servers, which performed real-time data quality control and weekly monitoring dashboards. Confidentiality of data and anonymity is guaranteed by Santé Publique France and its service provider Ipsos.

### Data Analysis

First, participation (participation rate, refusal, ineligibility) will be analyzed according to each intervention group, campsite, and data collection week in order to discuss potential selection bias.

When possible, logical tests between questions will be performed to identify possible comprehension problems.

To identify social and other determinants influencing sun protection behaviors from data collected before intervention at T0, we will use multivariate regression models. To do this, protection and exposure scores will have to be constructed from the different corresponding variables.

In order to evaluate the efficacy of the two interventions, we will first describe the knowledge, attitudes, intention to change and current behaviors of the tourists in each intervention group at T0 in order to ensure comparability of groups at inclusion. We will then compare the evolution of the main and secondary outcomes in the three groups (i.e., control group, intervention 1 group, intervention 2 group), using multivariate models for longitudinal data from T0–T1–T2 questionnaire data and T0–T1 skin color data. We will measure the evolution of the different psychosocial mechanisms (self-efficacy, perception of vulnerability, knowledge, social norms, and decisional balance) which comprise the interventions, as well as the influence of social characteristics.

All descriptive and analytical analyses will take into account the study design, the different cluster effects and the sample design. The analyses will be performed using Stata, R, and SAS software.

## Discussion

**Figure 3:**
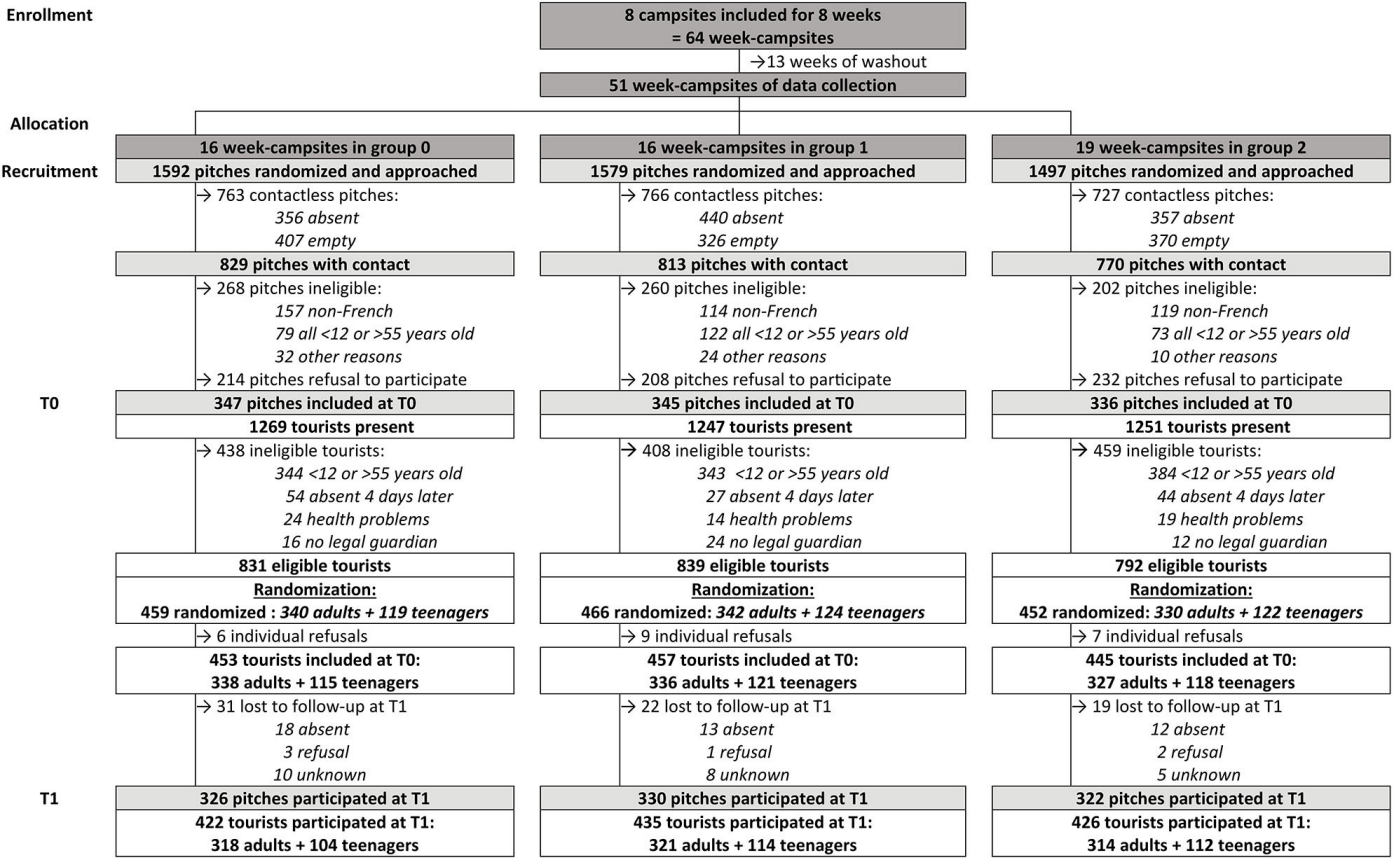
Flowchart describing participant recruitment and follow-up until T1—PRISME.

This first phase of the study (2019) included 1,355 participants (1,001 adults and 354 teenagers) on 1,028 campsite pitches (respectively 347, 345, and 336 pitches in each intervention group), reaching the calculated required sample size (328 pitches in each group) (Figure 3). Only 5% of participants were lost to follow-up at T1 (Figure 3) and only 3% refused to provide their email and were therefore inevitably lost to follow-up for the upcoming T2 in 2020. We are aware that online participation in this second follow-up will depend greatly on the quality and stability of the web interface as well as the attractiveness of the invitation to participate email and that there is a risk that this email as well as the information emails sent during the year will not be read.

In the first phase, we had to face several difficulties and limitations associated with our study. Specifically, the protocol's design was constrained by the fact that we had to interlink an epidemiological study to identify the determinants of sun protection with a randomized trial to evaluate the interventions.

The study's first aim required a closed environment, a countable and representative population of the entire tourist population with contrasting exposure and a detailed questionnaire. These criteria favored the choice of campsites as a study location rather than beaches.

The study's second aim required a sufficiently long intervention time to encourage exchanges with the participant, long-term follow-up, the support of tourism professionals, and interventions which could be implemented without using the campsite's human and financial resources. These needs led us to implement interventions focusing on individual-based prevention strategies, instead of more global interventions like modifying the physical environment, for example creating areas of shade. Indeed, after visiting the campsites, modifying the existing environment was considered expensive and difficult to implement and standardize.

Finally, the combination of these two aims resulted in a required participation time of at least 40 consecutive minutes (20 min for the questionnaire + 20–25 min for the intervention). This was often considered too long by the participants, and which may have led to a possible loss of attention.

In addition, our study took place in the context of short summer stays. This forced the first follow-up T1 to be very close to inclusion (3 or 4 days after), which may have been too short a period to measure changes in behavior. That is why we decided to also measure intention to change which could be considered an earlier predictor of future changes according to the transtheoretical model (17, 24).

Moreover, due to the seasonal nature of high-risk exposure situation during summer vacation, the second follow-up T2 could only be performed 1 year after the first follow-up. This may lead to memory bias at the end of summer 2020. Furthermore, unlike T0 and T1 which used a face-to-face questionnaire, data collection for T2 will use a web-based questionnaire and this may potentially affect answers.

We recommend to investigators of future similar studies to anticipate the progressive increase in the number of summer tourist stays in order to estimate the required weekly number of interviewers and participants as effectively and efficiently as possible. Indeed, at the start of the 2019 season, during the first 2 weeks of July, many of the campsite pitches we approached were empty (Table 1). This forced us to adapt the protocol by adding T0 data collection on Tuesday, something which was not initially scheduled, and to adapt the allocation of the intervention group in some campsites for certain weeks (Table 1). This also forced us to adapt the randomization method for choosing pitches by combining random selections from the list of existing pitches (potentially empty) and from booking lists (definitely occupied), which required us to regularly ask for booking lists from campsite owners throughout the summer. Despite these drawbacks, we reached the required target sample size. The potential selection bias induced by these protocol adjustments will be evaluated at a later date using sensitivity analyses.

The PRISME study has some important strengths. It is the first French study to compare a health-based sun-protection intervention with an appearance-based one. The protocol took into consideration several gaps identified in associated literature (16). Both interventions were theoretically constructed from two behavioral theories. Participants were of both sexes and varied in age from 12 to 55 years old. Protection of children under 12 years old was indirectly described by interviewing parents. People at particular risk, specifically people with personal or family antecedents of skin cancer, were not excluded if their sun exposure was not completely zero. This enabled us to evaluate the influence of this determinant. The required sample size, based on statistical power calculation, was reached. A second follow-up (T2) by web-questionnaire is planned for the end of the 2020 summer to evaluate possible long-term effects of both interventions on changes in sun-protection behaviors. The pre-study training of interviewers and prevention workers for 1–3 days by an experienced team and their supervision during T0 and T1 were also important elements to limit bias. Nevertheless, interviewer and prevention worker bias will still be taken into account in the analyses. In addition, our study is likely to suffer from a social desirability bias, with tourists reporting the behavior they think the researchers expects from them. Given this bias arising from self-reported data, PRISME included a measure of skin color using a colorimeter as one of the secondary outcomes in order to collect a more objective data (19, 25).

Support from tourism professionals was a major concern at the start of the project. Indeed, the successful implementation of the sun-protection interventions in a tourist setting depended on creating a strong partnership with tourism professionals who, naturally, may have been worried that the interventions' messages would negatively affect their future business, especially messages encouraging people to spend less time in the sun (11). We called them, then met them individually, presented the project positively and insisted on the fun of the interventions' activities, and the health value of increased sun protection. The pilot study was also an important step, not only to make changes to the questionnaire, but to enlist the collaboration of the first two campsites, and to reassure other campsites about the project's feasibility. All these exchanges helped to build trust and encourage tourism professionals' involvement.

Finally, this study is the first to evaluate an appearance-based sun-protection intervention in the French population. Although this approach has already shown promising results in studies on Anglo-Saxon populations, PRISME will help to analyze the efficacy and the transferability of this type of intervention to France, where social norms and the environmental context are different. By comparing the efficacy of a health-based intervention with an appearance-based one, our results may inform policy makers regarding prevention messages for tourists. Moreover, by studying participants' determinants, in particular social determinants, associated with sun protection and the intervention's effects, this study should lead to better identification of both at-risk groups and the mechanisms to activate, in order to influence a change of sun-protection behaviors in these groups during vacation. Depending on the results of PRISME, we will be able to contemplate designing future preventive campaigns and tools for the tourist population, especially using new technologies and images.

## Ethics Statement

### Ethics Approval

This study was an experiment in the human and social sciences in the health field. The study was approved by the Commission nationale informatiques et libertés (CNIL) (Decision DR-2019-110 of 25 April 2019 relating to request no. 919075) and by the Comité d'expertise pour les recherches, les études et les évaluations dans le domaine de la santé (CEREES) of the Institut national des données de santé (INDS) (TPS 303174, 14 February 2019).

### Information of Participants and Consent to Participate

The interviewers collected specific verbal consent from the participants (or a legal guardian for minors) prior to inclusion, and provided them with a detailed information letter including all their legal rights (for adults: Supplementary Material 6 and for miniors: Supplementary Material 7). Written informed consent was not required in accordance with the national legislation and the institutional requirements.

## Author Contributions

CDu was responsible for writing the protocol, implementing and monitoring the field study, data analysis, and the writing of this article. OC, DM, and CDe supervised this work and contributed to the writing of this article. OC participated in the campsite enrollment. AB and FC-G participated in the construction of the intervention, in the creation of the study material, in the recruitment and training of prevention workers, and in their field supervision. J-BR calculated the sample size, participated in the choice of the study design, the randomization method, and in field monitoring. M-LB participated in the choice of the colorimeter and the photo equipment and in the creation of study material. CM participated in the methodology and adaptation of questionnaires. EM was consulted during the writing of the protocol and participated in the creation of study material. All authors contributed to the article and approved the submitted version.

## Conflict of Interest

The authors declare that the research was conducted in the absence of any commercial or financial relationships that could be construed as a potential conflict of interest.

## Footnotes

^1^https://www.who.int/uv/faq/skincancer/en/index1.html

^2^https://www.insee.fr/fr/statistiques/4255862#consulter

^3^http://www.plferrer.photos/

^4^https://www.icm.unicancer.fr/fr/prevention/epidaure-pole-prevention

^5^http://www.delfintech.com/en/product_information/skincolorcatch

## Supplementary Material

Supplementary Material 1 Intervention booklet used in health-based intervention (intervention 1).

Supplementary Material 2 Intervention booklet used in appearance-based intervention (intervention 2).

Supplementary Material 3 Prevention summary record.

Supplementary Material 4 T0 questionnaire.

Supplementary Material 5 T1 questionnaire.

Supplementary Material 6 Information letter for adults.

Supplementary Material 7 Information letter for minors.

## Abbreviations

CEREES, The Expert Committee for Research: Studies and Evaluations in the field of Health
INDS: The National Institute of Health Data
CNIL: The French data protection authority
SPF: Sun protection factor
PRISME: PRevention and Impact of Sun exposure on the French MEditerranean coast
T0: Time 0 = inclusion
T1: Time 1 = first follow-up
T2: Time 2 = second follow-up
Nm: nanometer
ITA: Individual typology angle
CIE: color space defined by the International Commission on Illumination
RGB, Red, Green: Blue color model.
