# Supplementary material for: Effect of an Appearance-Based vs. a Health-Based Sun-Protective Intervention on French Summer Tourists' Behaviors in a Cluster Randomized Crossover Trial: The PRISME Protocol
Source: Front Public Health. 2020 Nov 5;8:569857. doi: 10.3389/fpubh.2020.569857 (PMC7676153; doi:10.3389/fpubh.2020.569857)
Supplement: Supplementary Material 2 — Intervention booklet used in appearance-based intervention (intervention 2). [file Data_Sheet_2.pdf]

MON CARNET SOLEIL

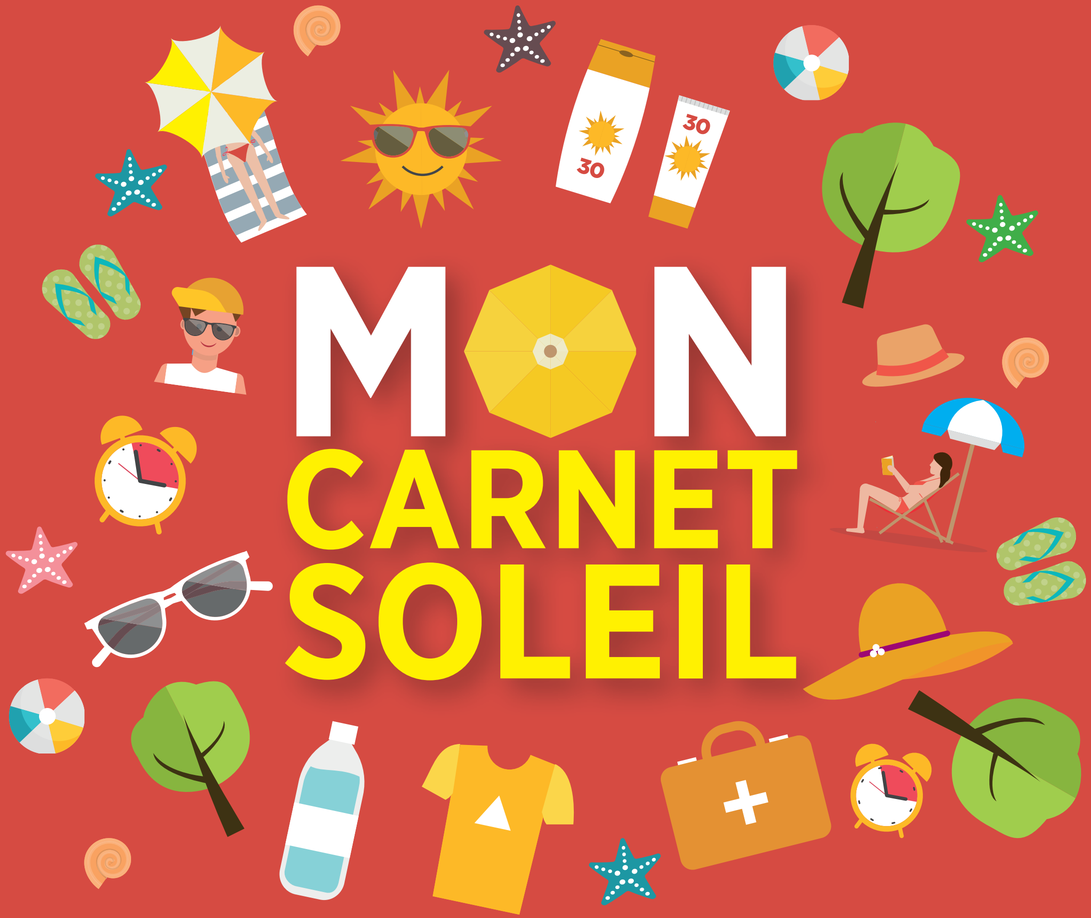

2019

Nom : ....

Prénom : ....

Ville : ....

ICM
Institut régional du Cancer
Montpellier | Val d'Aurelle

ars
Agence Régionale de Santé
Occitanie

Santé publique France

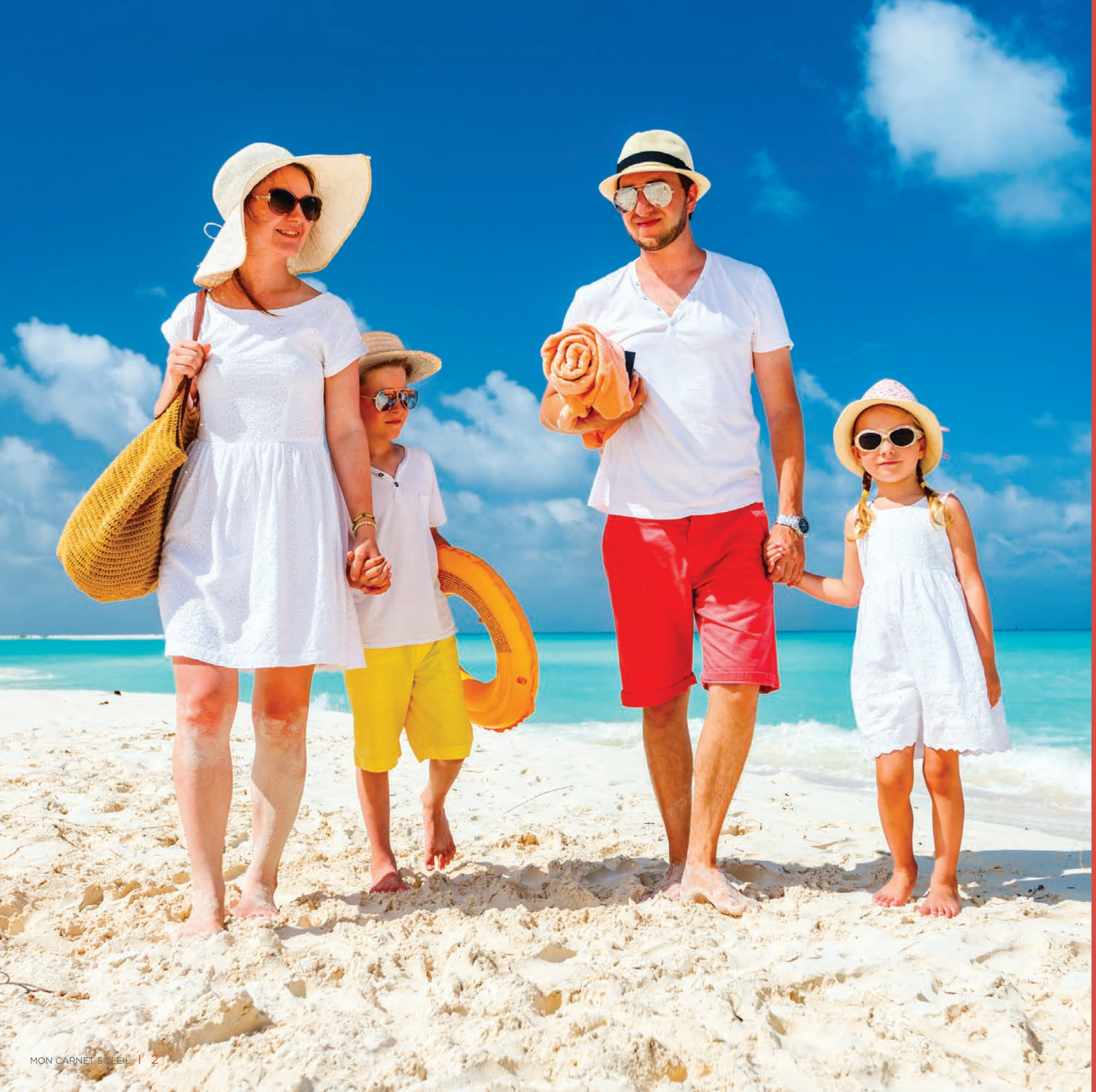

PRISME,
la protection solaire c'est capital !

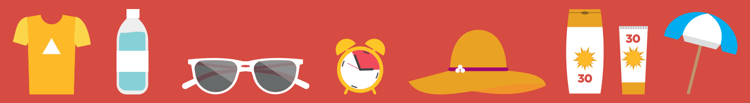

Lorsque nous partons en vacances nous souhaitons que la météo soit clémente et que le soleil soit au rendez-vous afin de profiter au maximum. Piscine, plage, barbecue, sorties, balades... sont bien plus agréables avec un beau ciel bleu.

En plus d'ensoleiller les vacances, le soleil est indispensable à la vie sur Terre et il est nécessaire à notre bonne santé.

Cependant, il faut savoir s'en protéger car une exposition inadaptée ou répétée entraine des effets sur notre santé (notamment cancers de la peau) ainsi que des effets inesthétiques visibles sur notre peau.
Prendre conscience plus particulièrement de ces effets sur l'apparence physique et apprendre à adopter les bons comportements face au soleil constituent l'objectif des activités qui sont proposées dans ce carnet.

Vous avez choisi de passer vos vacances dans un des campings du littoral méditerranéen.

Ce littoral est une zone de forte attraction touristique avec un fort rayonnement favorisant une exposition excessive au soleil.

Votre camping est inclus dans le projet PRISME
(Prévention et Impact de l'exposition Solaire sur le littoral MEditerranéen),
projet de recherche qui s'inscrit dans les objectifs de Santé Publique France.

Vous allez donc participer à une intervention sur la thématique prévention solaire.

Ce carnet servira de support à cette intervention.
A s'approprier et à personnaliser sans modération !

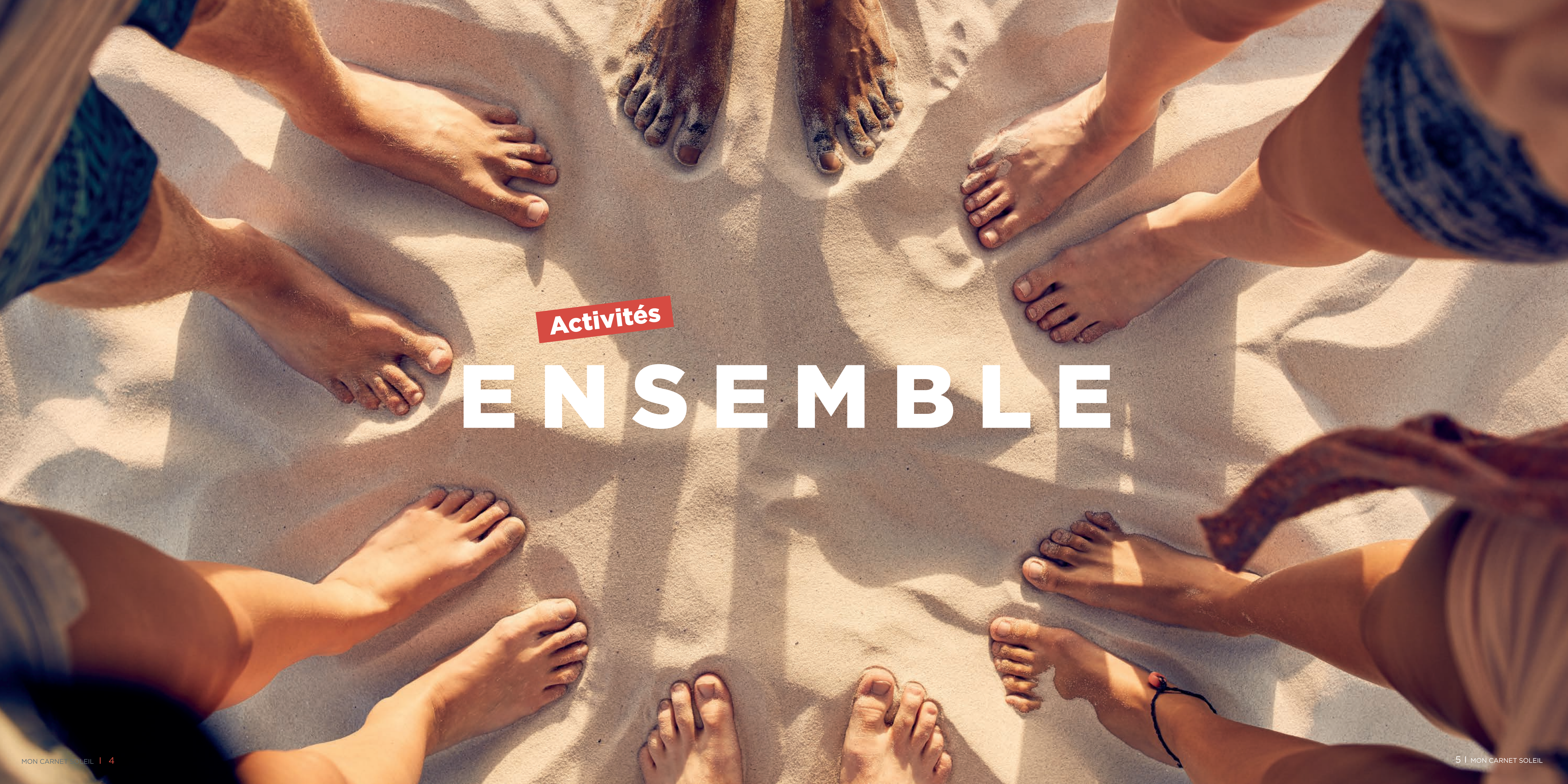

Activités

ENSEMBLE

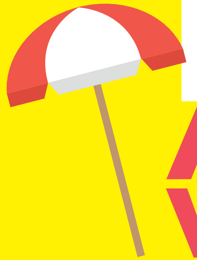

LES ACTIVITÉS

ACTIVITÉ 01

Quels sont les effets visibles du soleil sur mon apparence physique ?

Une exposition solaire excessive engendre un vieillissement cutané prématuré (ou photo vieillissement)

A savoir

Je connais les effets du soleil sur mon apparence physique

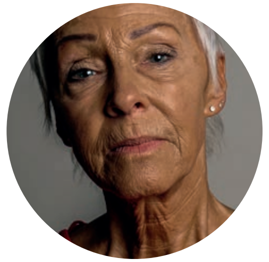

Bronzage
Epaississement et assèchement de la peau

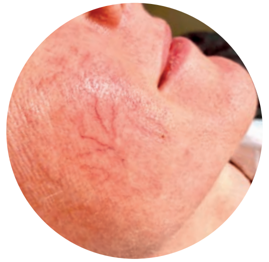

Atteintes des vaisseaux cutanés
Télangiectasies

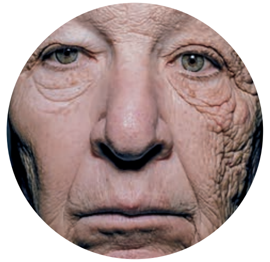

Perte d'élasticité
Relâchement cutané

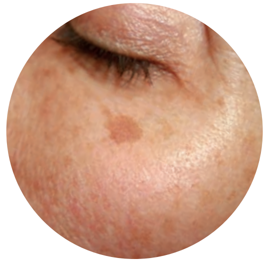

Apparition de taches brunes
Lentigos

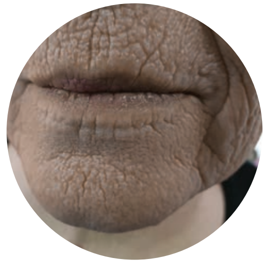

Accélération du vieillissement des cellules de la peau
Rides, ridules

Le photo-vieillissement est un vieillissement prématuré de la peau lié à l'exposition chronique ou intense au soleil et est différent du vieillissement normal de la peau dû à l'âge.

Le photo-vieillissement est irréversible mais ses conséquences sont essentiellement esthétiques, et non sanitaires.

Caractéristiques du photo-vieillissement

- Peau épaissie, rugueuse et sèche
- Dilatation des petits vaisseaux
- Peau lâche, perte d'élasticité
- Apparition taches brunes
- Ridules et rides profondes

ACTI VITÉ 02

Quels sont les effets non visibles du soleil sur ma peau ?

Je colle mes photographies UV prises avec l'animateur

Si des taches brunes sont visibles sur votre photo UV, elles représentent des dommages sur votre peau en grande partie liés à votre exposition solaire.
Plus vos taches sont nombreuses et grosses plus vos dommages liés au soleil sont importants.
Sans protection solaire, ces dommages vont continuer à croître jusqu'à devenir de plus en plus visibles et inesthétiques.

Pour limiter la progression de ces dommage cutanés, il est indispensable de se protéger du soleil. Des règles rapides et efficaces s'appliquent pour tous.

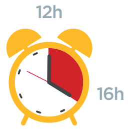

Éviter de s'exposer au soleil entre 12h et 16h

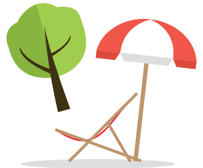

Rechercher l'ombre

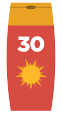

Renouveler toutes les 2h l'application de crème solaire *(indice 30 minimum pour les adultes et 50 pour les enfants)*

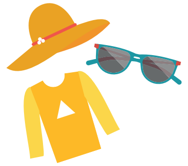

Sortir couvert *vêtements, chapeau, lunettes*

Se mettre à l'ombre et éviter le soleil entre 12h et 16h sont à privilégier. Si vous devez aller au soleil, utilisez vêtements et accessoires. La crème solaire complète la protection sur les parties découvertes.

ACTI VITÉ 02

Je compare ma photo UV aux photographies témoins

Les photos témoins représentent différents niveaux de photo-vieillissement. Elles vous aident à avoir des éléments de comparaison pour interpréter votre photo UV.

A savoir

L'appareil photo UV révèle des dommages solaires non visibles à l'œil nu. Ces dommages esthétiques ne sont pas des lésions précancéreuses mais sont les témoins d'une exposition solaire passée sans protection.

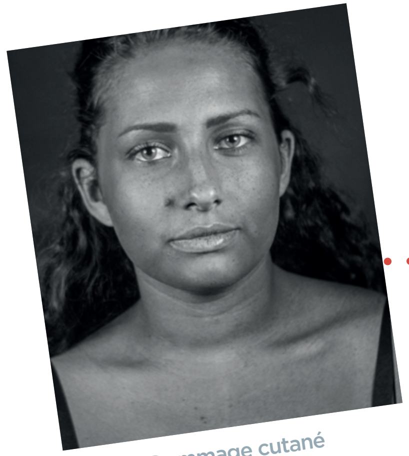

Dommage cutané léger

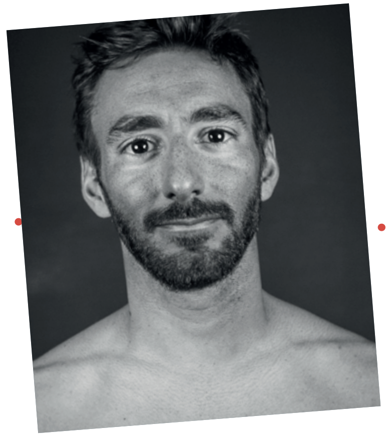

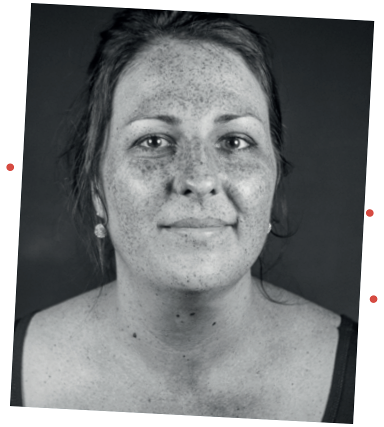

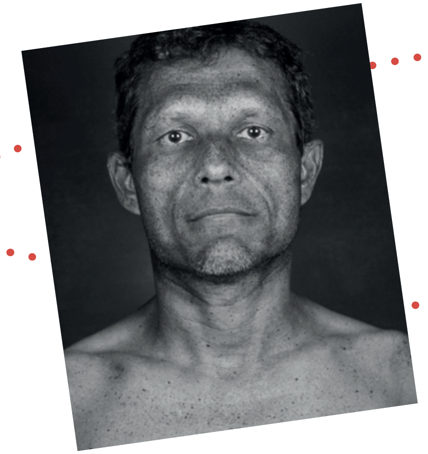

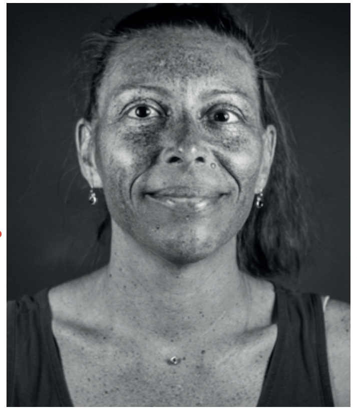

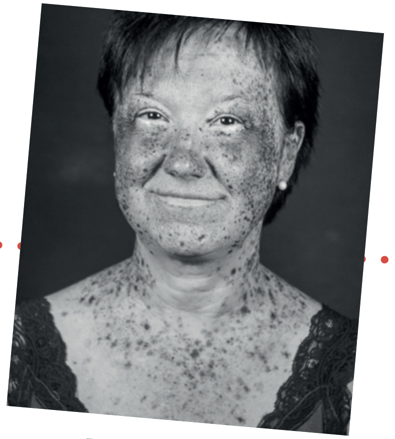

Dommage cutané sévère

Photos réalisées par Pierre-Louis Ferrer

ACTI
VITÉ
03

Quel est selon moi, l'idéal de bronzage ?

Je choisis la couleur de peau que je trouve la plus attrayante sur cette personne

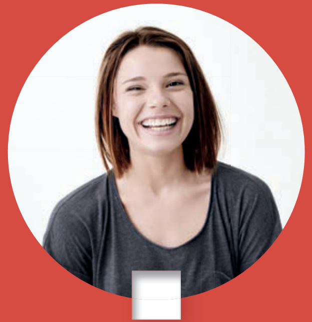
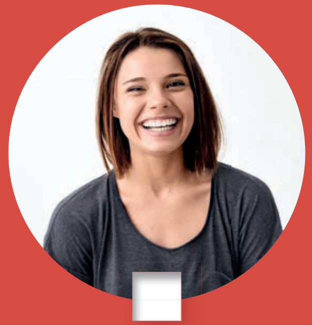
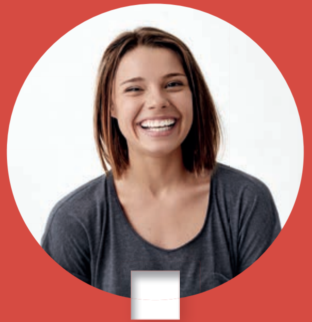
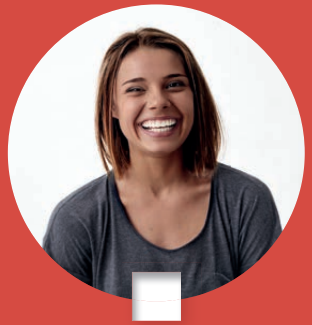
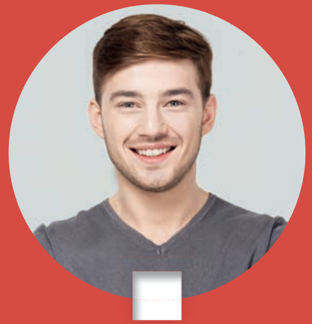
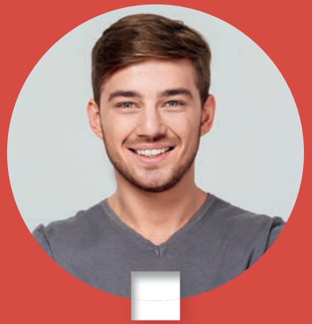
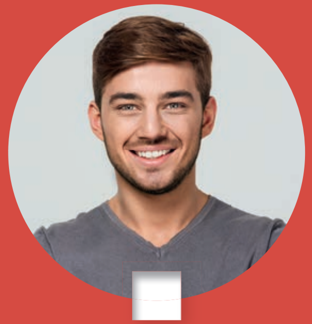
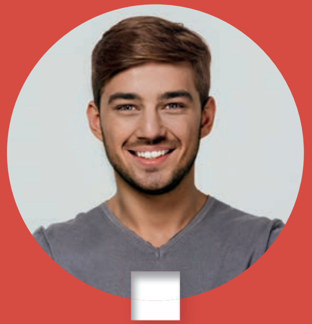

ACTI
VITÉ
03

Comment le bronzage est-il mis en valeur par les médias ?

Les médias valorisent le bronzage dans l'opinion publique et en font un synonyme de beauté et de bien être

A savoir

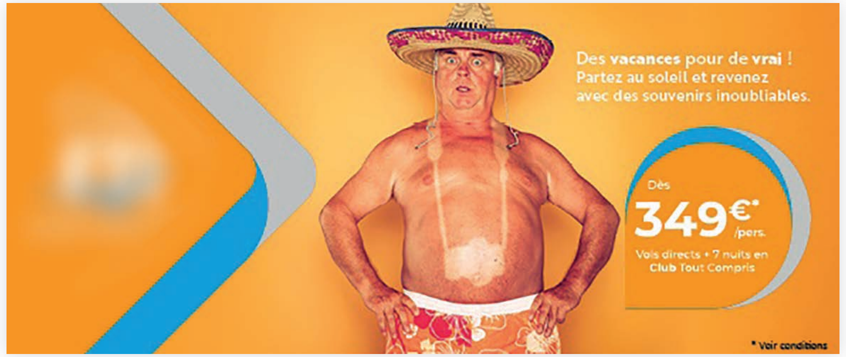

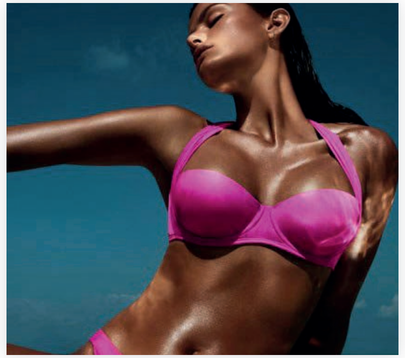

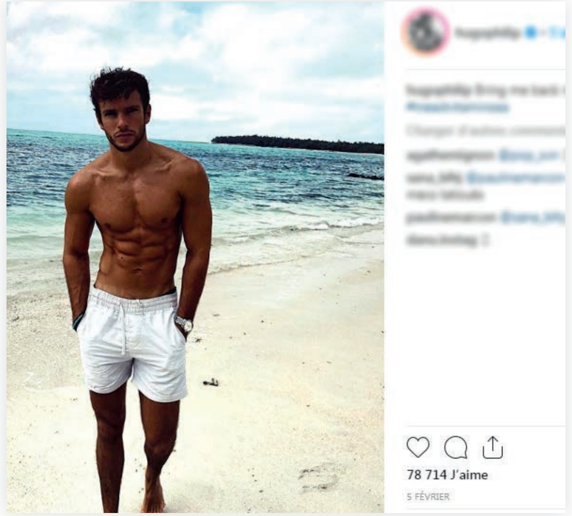

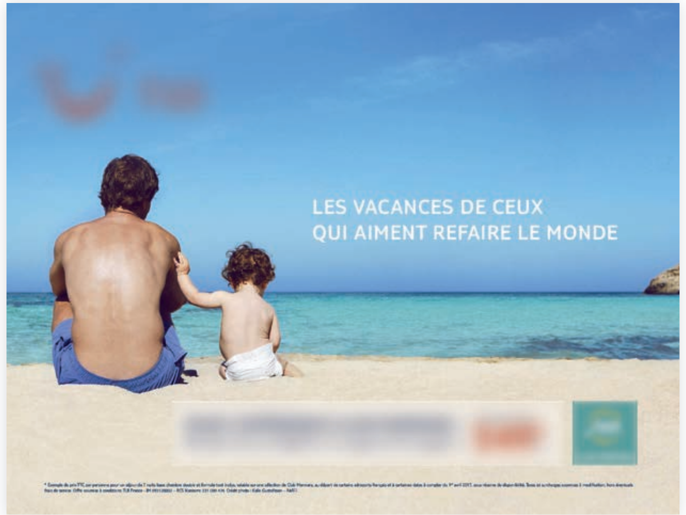

ACTI
VITÉ
04

J'imagine la suite de mes vacances au camping et je choisis une situation dans laquelle je pense être capable de me protéger du soleil

Quel comportement suis-je capable d'adopter pour me protéger du soleil ?

1

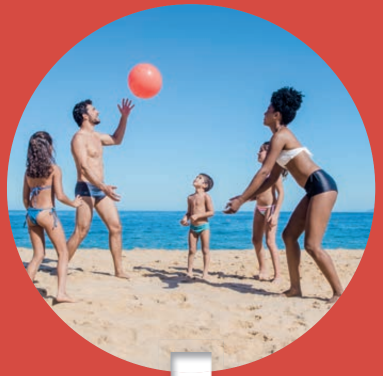

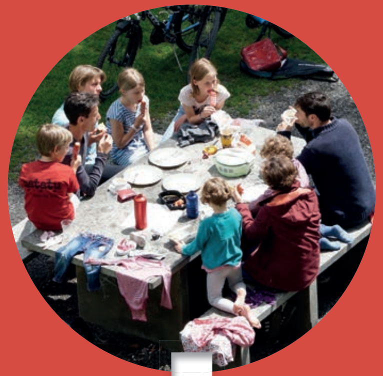

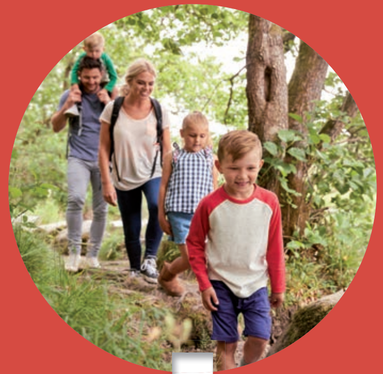

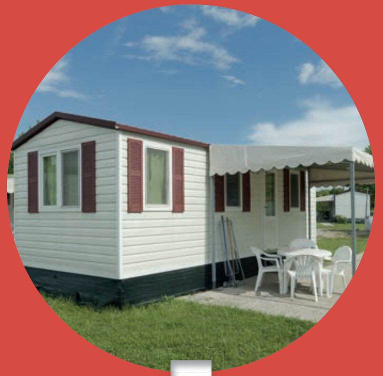

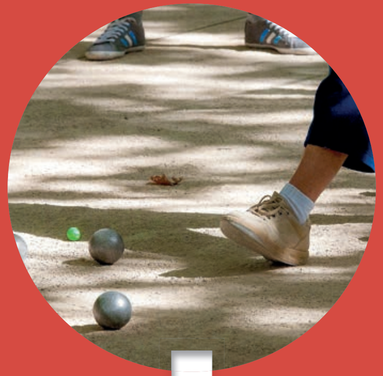

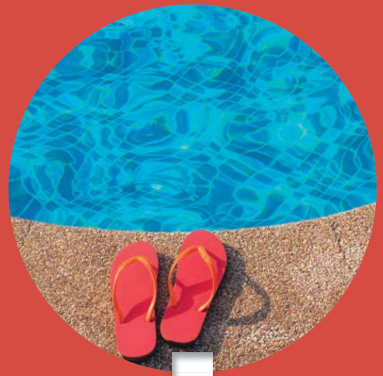

ACTI
VITÉ
04

je suis capable d'adopter le bon comportement dans différentes situations lors de mon séjour

A savoir

2

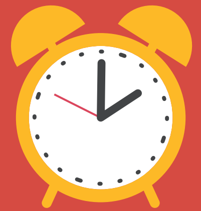

___ h ___

3

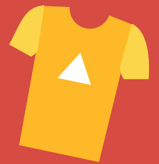

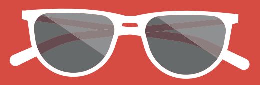

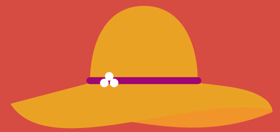

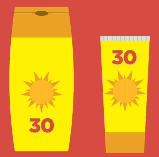

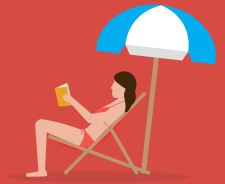

ACTI VITÉ 05

Quels sont les avantages et les inconvénients à l'utilisation des moyens de protection ?

Je coche la recommandation que j'ai le plus de mal à appliquer (voir activité 3 précédente)

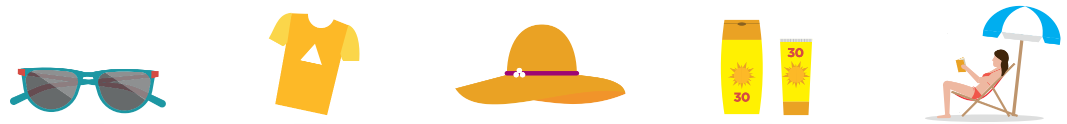

☐ ☐ ☐ ☐ ☐

Je note dans les cases ci-dessous les avantages et inconvénients pour chaque situation (voir activité précédente)

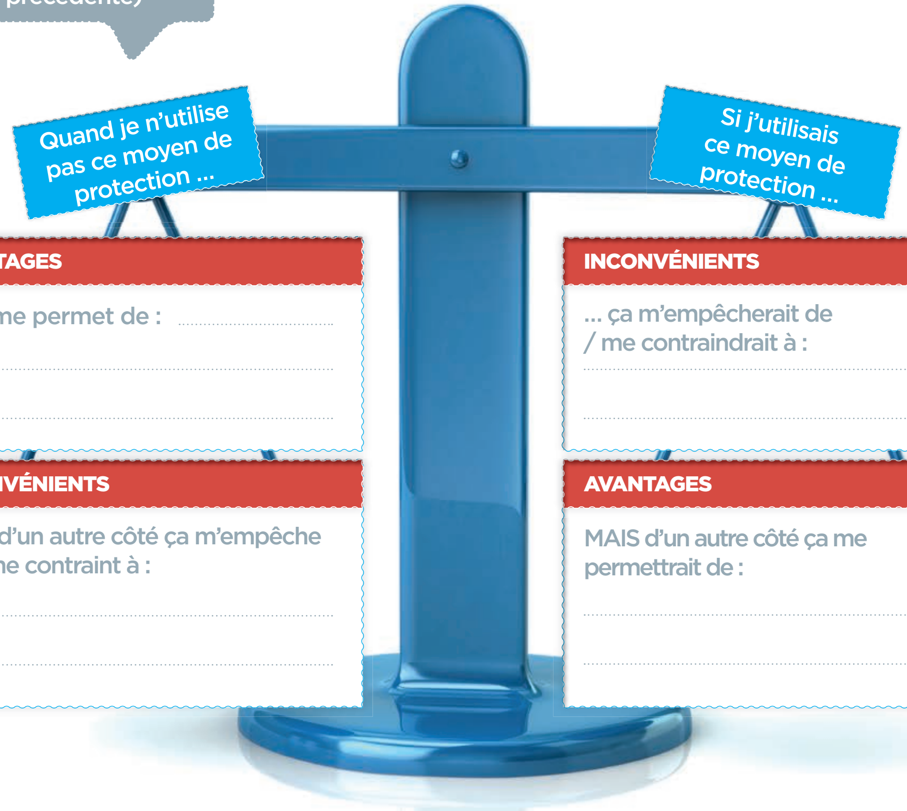

AVANTAGES

... ça me permet de : ....

INCONVÉNIENTS

MAIS d'un autre côté ça m'empêche de / me contraint à : ....

INCONVÉNIENTS

... ça m'empêcherait de / me contraindrait à : ....

AVANTAGES

MAIS d'un autre côté ça me permettrait de : ....

Pour compléter cette activité, vous trouverez page suivante des exemples de freins à la protection avec des solutions

ACTI VITÉ 05

Des exemples de freins à la protection

« Il n'y a pas d'ombre autour de moi »

Si vous êtes dans un endroit sans ombre le mieux est de rester à l'intérieur surtout entre 12h et 16h.

Si vous devez absolument sortir prenez avec vous un moyen de vous faire de l'ombre (parasol, ombrelle...) et des moyens de protection (t-shirt, chapeau, lunettes, crème solaire)

« Les produits solaires coûtent trop cher »

Nul besoin d'acheter la crème solaire la plus coûteuse pour être correctement protégé.

Le plus important est de regarder que l'indice soit au moins égal à 30 et d'en remettre toutes les 2h, après les baignades ou après une activité durant laquelle vous avez transpiré. Il faut également en mettre en quantité suffisante pour être efficace (environ 1/4 de tube pour tout le corps d'un adulte). De plus, elle doit être utilisée en complément des autres moyens de protection sur les zones qui ne peuvent être protégées par un vêtement.

« Je n'ai jamais eu de coup de soleil »

Le danger ne vient pas uniquement des coups de soleil. Le bronzage est déjà un premier signe d'agression de la peau.

Les gens qui n'ont pas de coups de soleil sont également à risques. En effet, il existe 2 types d'UV (Ultraviolet) : les UVB et les UVA. Les UVB sont responsables du bronzage et des coups de soleil. Les UVA sont invisibles et pénètrent en profondeur. Les deux types d'UV sont dangereux pour la santé.

« J'ai une peau foncée »

Les personnes avec un profil peu sensible au soleil ont tout de même des risques. Elles doivent donc se protéger de manière efficace même si elles ont peu de coups de soleil.

« Je prends des compléments alimentaires pour préparer ma peau au soleil »

Ils donnent un teint hâlé mais aucun complément alimentaire ne protège du soleil car ce sont souvent de simples colorants (carotène).

« Je préfère mettre de la crème solaire et rester torse nu car l'été il fait très chaud »

La crème solaire ne protège pas à 100%, « l'écran total » n'existe pas. Même une crème solaire indice 50 ou 50+ laisse passer une partie des rayons UV. Elle doit être combinée avec les autres moyens de protection.

« Je suis à l'ombre, je n'ai pas besoin de mettre un t-shirt, des lunettes, un chapeau et de la crème solaire »

Tout comme la crème solaire l'ombre ne protège pas à 100% et certains UV sont réfléchis par le sol. Il est donc important de l'associer aux autres moyens de protection (chapeau, lunettes, t-shirt, crème) surtout entre 12h et 16h.

« Lorsqu'il fait un ciel gris et/ou du vent je n'ai pas envie de me protéger, j'ai l'impression que c'est inutile car il ne fait pas chaud »

Il est très important de se protéger quand il y a du vent ou des nuages car la chaleur n'est pas ressentie mais les UV traversent les nuages et ne sont pas atténués par le vent.

« Lorsque je me déplace ou lorsque je cours je n'ai pas besoin de me protéger »

Les rayons du soleil atteignent la peau de la même manière que l'on soit immobile ou en déplacement. Il est donc important de se protéger de manière efficace même lorsque l'on se déplace.

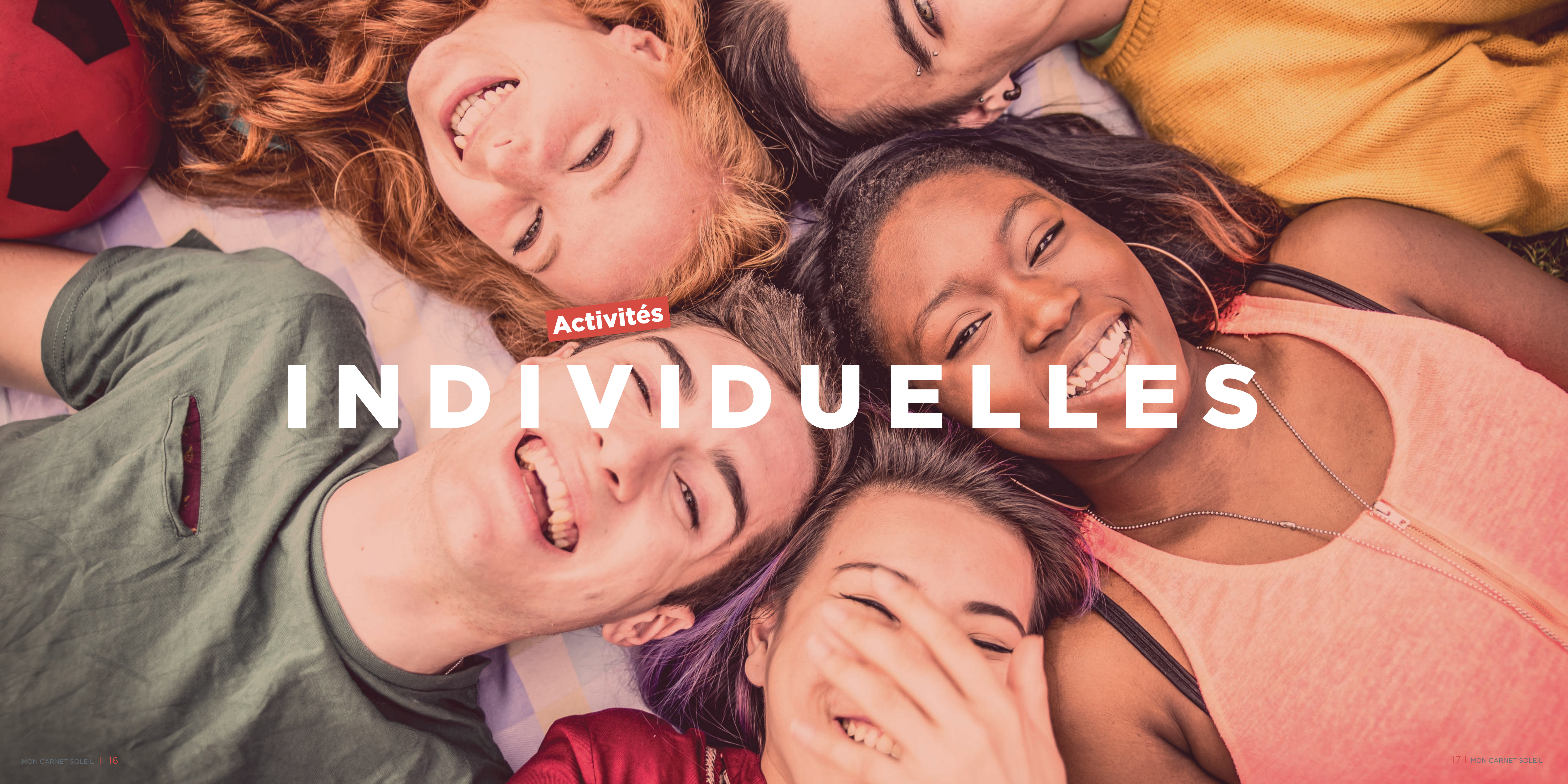

Activités

INDIVIDUELLES

Quel rôle je joue vis-à-vis de mon enfant ?

A savoir

N'exposez pas un enfant de moins de 3 ans directement au soleil

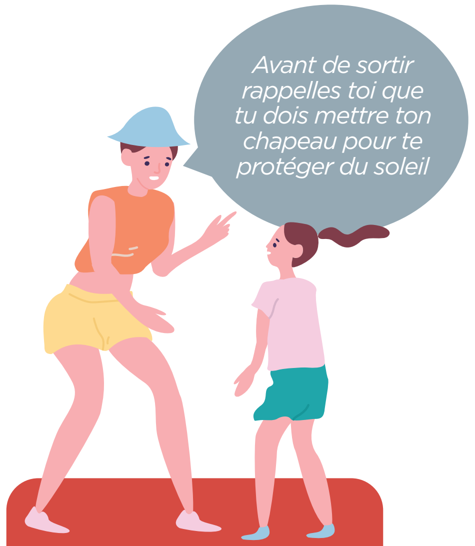

Je lui apprends à se protéger lui-même du soleil et à acquérir les bonnes habitudes

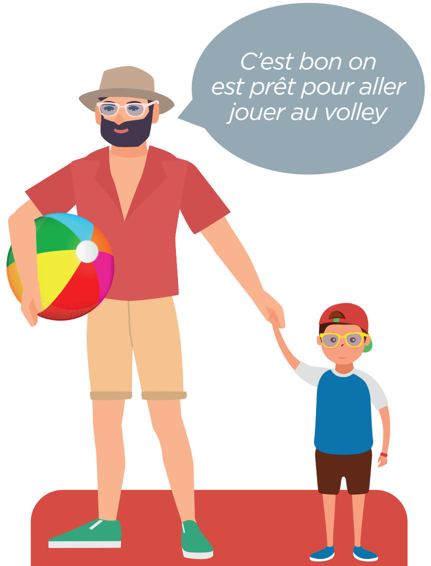

Je suis un modèle pour mon enfant, si je me protège il aura tendance à se protéger

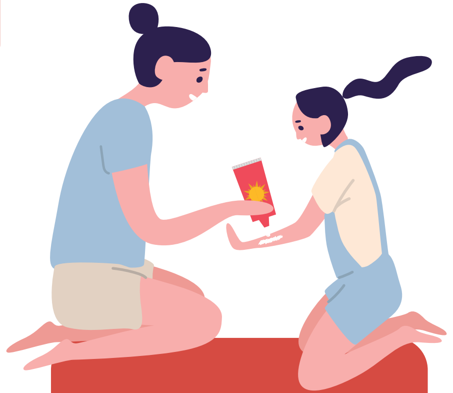

Je protège mon enfant en lui mettant les moyens de protection

Que puis-je faire entre 12h et 16h pour éviter de m'exposer ?

A savoir

L'été, le fait d'éviter de sortir entre 12h et 16h est la première des protections

- Je lis, je pratique un sport en salle, je vais à la piscine intérieure.
- Je me repose afin d'être en forme pour ne sortir qu'après 16h
- Je joue à des jeux de société ou je fais des activités manuelles à l'intérieur ou à l'ombre
- Je vais me balader dans des lieux ombragés (forêts)
- Je mange et je discute avec ma famille ou mes amis à l'ombre
- Je vais au cinéma, au musée, au bowling, à l'aquarium ou faire un escape game ...
- Je fais du shopping
- Je cuisine
- Je fais les activités à l'intérieur ou à l'ombre proposées par le camping (flipper, billard, jeu de palet, spa...)

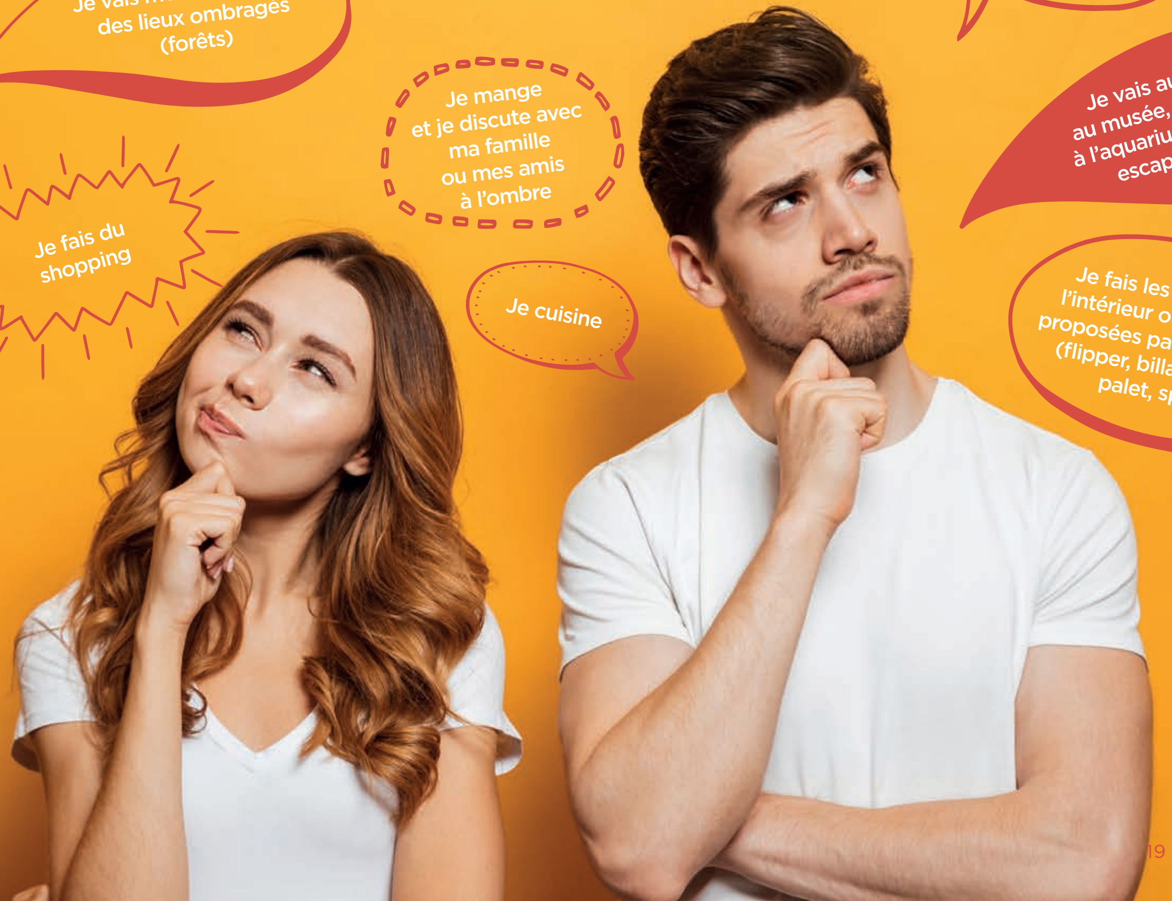

Que sont **les UV** et **l'indice UV** (Ultraviolet) ?

Composition du rayonnement

Ultraviolets	Lumière blanche visible	Infrarouges
Invisibles, ne chauffent pas	*composée de toutes les couleurs*	*Invisibles, donnent la sensation de chaleur*

Le rayonnement solaire est composé de la lumière visible (lumière blanche), de rayons infrarouges (qui sont invisibles et qui provoquent la sensation de chaleur) et de rayons UV (qui sont invisibles et qui ne provoquent pas de chaleur).

A savoir

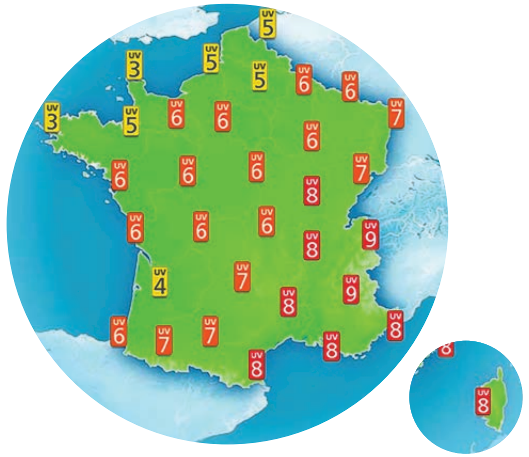

Figure : Exemple de carte de France d'indice UV du 29 juin 2016.
Source : www.meteofrance.com

L'indice UV reflète l'intensité du rayonnement UV du soleil et le risque qu'il représente pour la santé. Il varie selon le moment de la journée, selon les saisons et aussi selon la géographie.

A savoir

Attention :
Nous ne sentons pas les UV, ils peuvent être présents même si nous n'avons pas chaud ou par temps nuageux.

Indice UV	Intensité d'exposition	Protection recommandée
0	Nulle	Pas de protection nécessaire
1,2	Faible	Pas de protection nécessaire
3,4,5	Modéré	T-shirt, chapeau, lunettes, crème solaire
6,7	Elevée	Eviter le soleil entre 12h et 16h,si vous sortez : rechercher l'ombre, mettre T-shirt chapeau,lunettes, crème solaire
8,9,10	Très élevée	Eviter si possible tout séjour en plein air surtout entre 12h-16h
11 et +	Extrême	Eviter si possible tout séjour en plein air surtout entre 12h-16h

Tableau : Intensité d'exposition en fonction de l'indice UV ; Source : OMS

Attention :
À la plage ou au camping, le sable et l'eau réfléchissent les rayons UV ce qui nécessite d'être vigilant même sous un parasol ou dans l'eau.

Pour trouver l'indice UV jour après jour : www.soleil.info.fr ou www.meteofrance.com

Jeu du soleil

J'observe les deux images et je retrouve les 8 différences

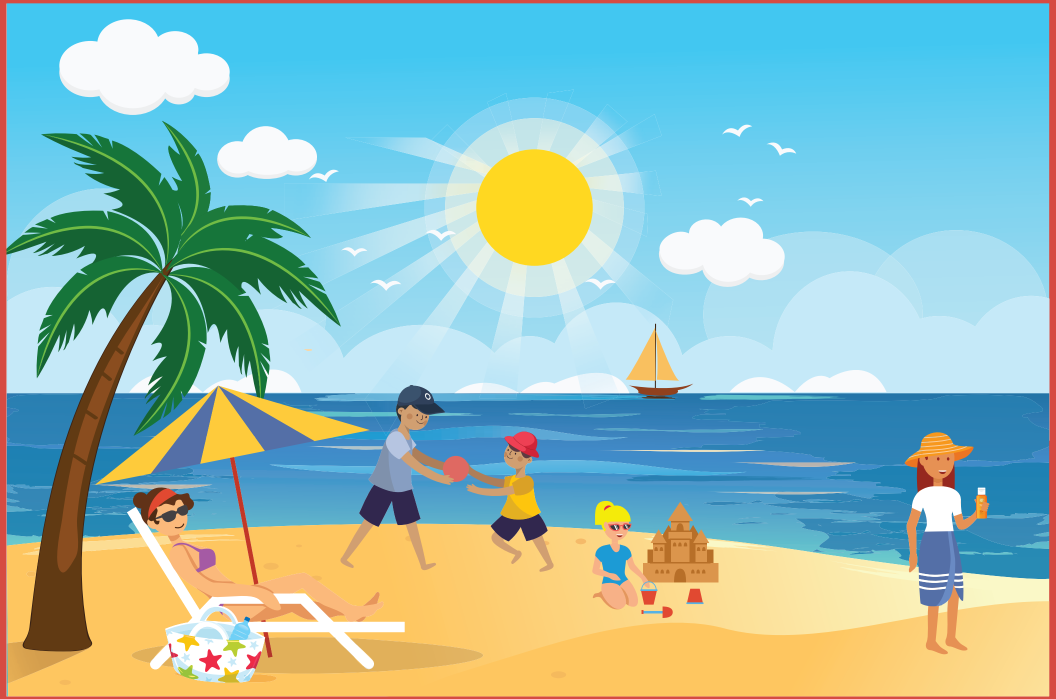

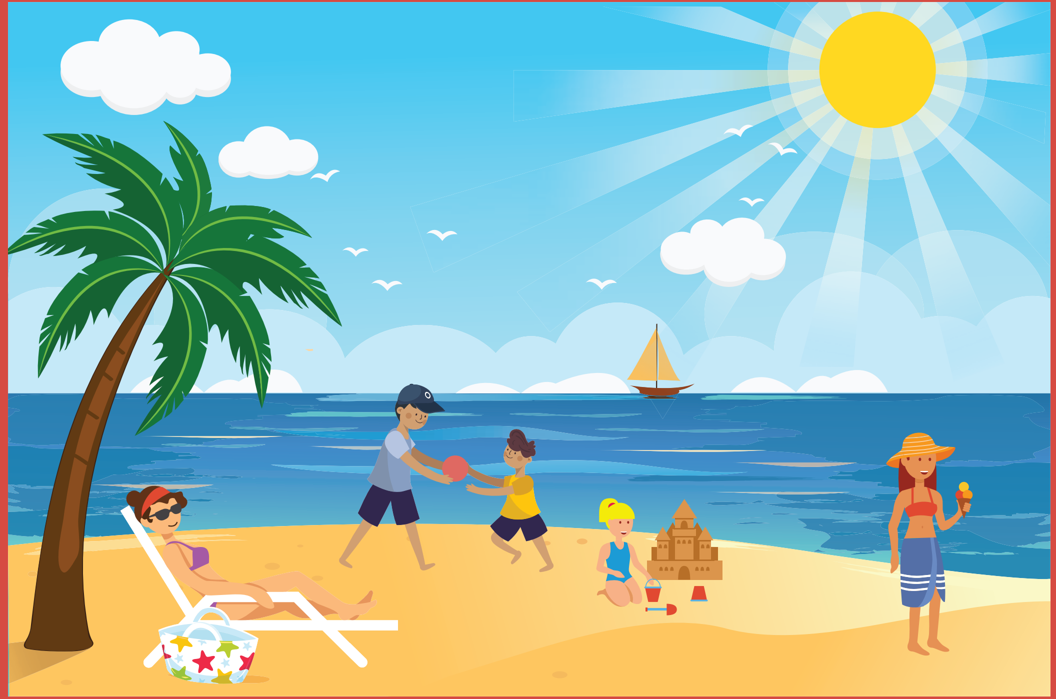

Solution :
Position du soleil, parasol à côté de la dame sous le palmier, bouteille d'eau dans le panier, chapeau sur l'enfant qui joue au ballon, lunettes et t-shirt de la fille qui construit un château de sable, crème solaire et un t-shirt pour la dame complètement à droite.

Quizz du soleil

Pour tester vos connaissances sur le soleil et les moyens de vous en protéger, remplissez ce quizz.

Question	Réponse	Explication
**1.** Nous sommes tous sensibles au soleil	☐ Vrai ☐ Faux	Vrai. Nous sommes tous sensibles au soleil mais certaines peaux le sont encore plus, notamment les peaux claires et celle des enfants et adolescents.
**2.** Sous un parasol pas besoin de se protéger	☐ Vrai ☐ Faux	Faux. Lorsque les rayons du soleil arrivent sur le sable ils « rebondissent » et peuvent donc nous toucher même sous le parasol. Cela s'appelle la réverbération du soleil.
**3.** La crème solaire suffit pour être bien protégé	☐ Vrai ☐ Faux	Faux. La crème solaire intervient en complément des autres moyens de protection (t-shirt, chapeau, lunettes). Elle doit être appliquée sur les parties découvertes, en quantité suffisante, renouvelée toutes les deux heures et après la baignade.
**4.** Les ultraviolets sont des rayons invisibles émis par le soleil	☐ Vrai ☐ Faux	Vrai. Les ultraviolets sont invisibles comme les infrarouges, mais à la différence de ces derniers, ils ne provoquent pas de sensation de chaleur. Même s'il fait frais ou s'il y a des nuages, les UV passent (l'index UV peut être élevé, pensez à consulter la météo).
**5.** On peut prendre un coup de soleil sur l'oeil	☐ Vrai ☐ Faux	Vrai. Tout comme pour la peau, les rayons solaires peuvent entraîner des « coups de soleil » ou brû-lures à la surface de l'oeil, on appelle cela l'ophtalmie. Il est donc important de porter des lunettes de soleil et un chapeau à bords larges (ou une casquette) pour se protéger les yeux. Préférez des lunettes couvrant bien les yeux et portant la norme CE3 ou CE4.
**6.** Le coup de soleil finit par s'en aller	☐ Vrai ☐ Faux	Vrai et faux. Le coup de soleil est un signal d'alerte suite à une exposition excessive aux UV. Il finit par s'estomper dans les jours suivants, cependant il montre qu'il y a eu des dégâts au niveau de la peau, bien souvent irréversibles.
**7.** Le soleil aide à fabriquer de la vitamine D	☐ Vrai ☐ Faux	Vrai. De courtes expositions au soleil (5-10 minutes), 2 à 3 fois par semaine par une journée ensoleil-lée) d'une petite partie du corps (visage et avant-bras) suffisent à maintenir un niveau satisfaisant de vitamine D.
**8.** Avec la crème solaire je peux m'exposer plus longtemps	☐ Vrai ☐ Faux	Faux. Aucune crème solaire ne filtre totalement les ultraviolets du soleil. Une partie des UV réussit malgré tout à traverser la peau et lorsqu'on augmente l'exposition au soleil, on augmente la quantité d'UV reçus.
**9.** En été c'est à midi que le soleil est le plus fort	☐ Vrai ☐ Faux	Faux. C'est à 14h que le soleil est le plus haut dans le ciel, on appelle ça le « midi solaire ». C'est à ce moment-là que notre ombre est la plus courte.
**10.** A la montagne en hiver, je n'ai pas besoin de me protéger du soleil	☐ Vrai ☐ Faux	Faux. L'index UV augmente avec l'altitude. Le risque de coups de soleil existe aussi en montagne. Il est amplifié par la réverbération des rayons UV sur la neige.

RESULTATS DU TEST

Faites votre total de bonnes réponses sur 10

/ 10

Notes personnelles

Points importants

Il faut savoir que se **mettre à l'ombre** et **éviter le soleil entre 12h et 16h** (horaires durant lesquels notre ombre est plus petite que nous) sont à privilégier en termes de protection solaire.
Ensuite pour compléter la protection il est important d'utiliser **vêtements et accessoires adaptés** (lunettes, t-shirt, chapeau/casquette). **La crème solaire** finalise cette protection, elle peut être appliquée sur les parties découvertes. Elle doit être minimum indice (SPF) 30 pour les adultes et 50 pour les enfants.

Une protection solaire optimale associe donc toutes ces recommandations.

Conseils prévention

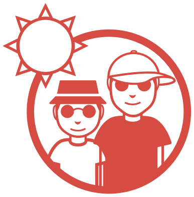

Peaux jeunes
= peaux sensibles

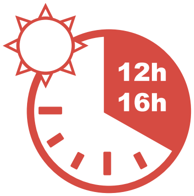

Évitez le soleil
de 12h à 16h

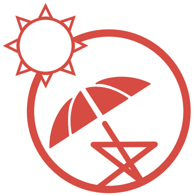

Recherchez l'ombre

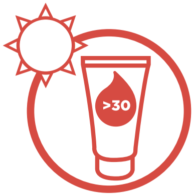

Renouvelez souvent
votre protection solaire

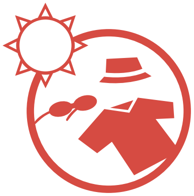

Couvrez-vous

Pour en savoir plus **www.prevention-soleil.fr**

ICM Institut régional du Cancer Montpellier | Val d'Aurelle

ars Agence Régionale de Santé Occitanie

Santé publique France

Mai 2019 - Crédits photos : ICM tous droits réservés, shutterstock.

JEROMEDEGUINES STUDIO DE CRÉATION FREELANCE
