# Supplementary material for: Effect of an Appearance-Based vs. a Health-Based Sun-Protective Intervention on French Summer Tourists' Behaviors in a Cluster Randomized Crossover Trial: The PRISME Protocol
Source: Front Public Health. 2020 Nov 5;8:569857. doi: 10.3389/fpubh.2020.569857 (PMC7676153; doi:10.3389/fpubh.2020.569857)
Supplement: Supplementary Material 3 — Prevention summary record. [file Data_Sheet_3.pdf]

PRISME				Prevention summary				
Prevention worker name :								
Date	Campsite	Intervention Group	Campsite pitch	Adult / Teenager	photo 1 number	photo 2 number	phototype	**Observation**<br>*(problems of adherance to protocol, feedback, intervention tool accuracy…)*
