# Supplementary material for: Effect of an Appearance-Based vs. a Health-Based Sun-Protective Intervention on French Summer Tourists' Behaviors in a Cluster Randomized Crossover Trial: The PRISME Protocol
Source: Front Public Health. 2020 Nov 5;8:569857. doi: 10.3389/fpubh.2020.569857 (PMC7676153; doi:10.3389/fpubh.2020.569857)
Supplement: Supplementary Material 4 — T0 questionnaire. [file Data_Sheet_4.pdf]

Questionnaire T0

Campsite Pitch questionnaire

Identification of site		
Name of campsite		
Number of pitch		
Intervention group	0/1/2	
Date and time of interview		
Interviewer's identification code		
Is the main residence of all the people present on the campsite pitch abroad?	Yes/No/DNK/Refusal to answer	
Has anyone currently staying on this campsite pitch already been asked in previous weeks to take part in this survey?	Yes/No/DNK	
Persons present at the campsite pitch :<br>Under 12 years old<br>12-17 years old<br>18-55 years old<br>>55 years old	_<br>_<br>_<br>_	
**For each person present aged between 12 and 55 years old**	**For those between 12 and 17 years old**	**For those between 18 and 55 years old**
Family Name – First Name		
Will this person still be at this campsite pitch in 4 days' time?	Yes/No/DNK/Refusal to answer	Yes/No/DNK/Refusal to answer
Has this person a health condition which totally prevents him/her from having any exposure to the sun?	Yes/No/DNK/Refusal to answer	Yes/No/DNK/Refusal to answer
Is this person accompanied by at least one of his/her legal guardians (parents or tutors)?	Yes/No/DNK/Refusal to answer	
	Selection of an adolescent between eligible 12- to 17-year olds.	***Selection of an adult between eligible 18- to 55-year olds.***

Information about the family household	
Do the two people selected live in the same household during the rest of the year?	Yes/No/DNK/Refusal to answer
**If answer is "No", or if only 1 person is interviewed**<br><br>**If answer is "Yes"**	Ask the following questions separately to each individual<br><br>Ask the following questions to only one person (Response used as response for the entire household)
Does the person in the household with the highest income practice a profession?	1. Yes<br>2. No, he/she is unemployed<br>3. No, he/she is retired<br>4. No, he/she is disabled<br>5. No, he/she is looking for his/her first job<br>6. No, he/she is a homemaker or has no profession<br>7. No, he/she is a student/college goer

	8. No, other reason	
**If answer is Practicing a profession (1)/ Unemployed (2) / Retired (3)**		
What is the profession of the person in the household with the highest income?		
Is he/she an employee or self-employed?	1 employee<br>2 self-employed<br>3 DNK	If answer is "1", is he/she a French public sector employee (whether at State or local/regional level), or an employee of a private organization?
		If answer is "2", how many employees does his/her firm have?<br>0-9 employees / 10 employees or more
What is his/her work qualification?	Unskilled worker / Skilled worker / Employee / Technician / Supervisor / Manager or engineer	
Socio-professional category (calculated) : 42 categories		
Socio-professional category (calculated) : 8 categories	Farmer / Craftsperson, trader, business manager / Senior manager / Intermediate professional / Employee / (Un)skilled worker / Retired / Inactive	
Socio-professional category (calculated) : 5 categories	Farmer / Craftsperson, trader, business manager, senior manager / Intermediate professional, employee / (Un)skilled worker / Retired , inactive	
Which county do you live in?	List of counties in France	
Are the arrival and departure dates for the campsite the same for both people?	Yes/No/DNK/Refusal to answer	
**If answer is "No", or if only 1 person is interviewed**<br><br>**If answer is "Yes"**	Ask the following questions separately to each individual<br><br>Ask the following questions to only one person (Response used as response for the entire household)	
What was the arrival date to the campsite?		
How long is/are the person/s staying?		
What is the type of accommodation?	Tent on bare pitch / Caravan / Campervan / Motorhome / Mobile home, chalet / Other	

Individual Questionnaire

Interviewee identification	
Participant's unique identification code	campsite number – pitch number – week number – individual's number
Family Name	
First name	
Email address	
If the minor does not have an email address or if the parents object to the minor's email address being given Email address of parent or legal guardian	
Telephone number	
If the minor does not have a telephone or if the parents object to the minor's telephone number being given Telephone number of parent or legal guardian	

Socio-demographic and socioeconomic characteristics		
Age	__	
Sex	Man / Woman	
**If a minor,**		
Which of your parents is with you during your stay here?	Your father (or tutor) /Your mother (or tutor)/Both	
What is your parents' highest educational diploma?	a) Father<br>No diploma/ Less than High school Diploma/High school diploma + 1 year / +2 years / + 3 years / +4 years / +5 years/ + 6 years and more of college	b) Mother<br>No diploma/ Less than High school Diploma/High school diploma + 1 year / +2 years / + 3 years / +4 years / +5 years/ + 6 years and more of college
**If an adult,**		
What is your highest educational diploma?	No diploma/ Less than High school Diploma/High school diploma + 1 year / +2 years / + 3 years / +4 years / +5 years/ + 6 years and more of college	
In your professional career, has your work ever involved you having to regularly work in the sun?	Yes/No/DNK/Refusal to answer	
During your childhood and adolescence, in which county in France or in which country did you live for the most time?	List of counties in France + list of foreign countries<br>*Several choices*	

Physical characteristics (self-reported phototype)	
What is the color of your skin without a tan on unexposed areas of your body (for example, the underside of your arm near the armpit)?	Very light or white / Light or pale / Quite light to slightly golden / Light brown, matte, olive / Dark brown / Black
What color are your eyes?	Light blue / light grey / light green / Blue / green / grey / Light brown or hazel / Dark brown / Brown-Black
What is the natural color (without any coloring/dyeing) of your hair?	Red or light blond / blond / dark blond or light brown / chestnut / dark brown / Black
Do you have freckles and moles on unexposed areas of your body?	Many / Several / Few / Very few / None

If you were exposed to the sun for the first time in the year in early summer, without any protection, for 1 hour in the middle of the afternoon, how would your skin react to the sun the next day?	I would always / often / sometimes / rarely / never get sunburned
If you were exposed to the sun for the first time in the year at the beginning of summer, without any protection, for 1 hour in the middle of the afternoon, how would your skin react to the sun a week later?	I would have no tan / a light tan /a medium tan / a dark tan
Heath characteristics	
Have you ever been or are you currently being treated or followed for skin cancer?	Yes/No/DNK/Refusal to answer
Has a loved one, family member, friend or colleague had skin cancer?	Yes/No/DNK/Refusal to answer
Did you have (adultes) / Have you had (adolescents) severe sunburn, that is to say with blisters or peeling skin, during childhood or adolescence?	Yes, once / Yes, several times / No, never / I don't remember / Refusal to answer
Do you have any health problem for which a doctor currently has:	Recommended you spend time in the sun (psoriasis, significant deficiency in Vit D, etc.)<br>/ Recommended you do not spend time in the sun (pregnancy, medically photosensitive skin , history of skin cancer, allergy to the sun, immunosuppression, etc.)<br>/ No, neither of the 2 options

Knowledge	
In your opinion, what are the ways to protect oneself from the sun?	Stay in the shade / Wear sunglasses / Wear a t-shirt / Wear a hat or cap / Put on sunscreen / Avoid the sunniest hours / Other / DNK / Refusal to answer<br><br>*Do not list answers*<br>*SEVERAL ANSWERS POSSIBLE*
In your opinion, in the summer in France, what are the most dangerous times of the day when it is better not to stay in the sun?	Before 9 a.m. / 9 a.m. to 10 a.m. / 10 a.m. to 11 a.m. / 11 a.m. to 12 p.m. / 12 p.m. to 1 p.m. / 1 p.m. to 2 p.m. / 2 p.m. to 3 p.m. / 3 p.m. to 4 p.m. / 4 p.m. to 5 p.m. / 5 p.m. to 6 p.m. / 6 p.m. to 7 p.m. / 7 p.m. to 8 p.m. / After 8 p.m. / None / DNK / Refusal to answer<br><br>*Do not list answers*<br>*SEVERAL ANSWERS POSSIBLE*
Without doing any specific physical activity or swimming, how often should a person put on sunscreen while in the sun?	Every 30 mins / Every hour / Every 2h / Every 3h / Every 4h / Once during the day / DNK / Refusal to answer
What do you think are the possible consequences on the body of intense exposure to the sun, immediately after and in the longer term?	Sunburns, burns<br>/ Sunstroke, dehydration, heat stroke<br>/ Skin rashes, pimples, sun allergies<br>/ eye problems, conjunctivitis, eye inflammation, photokeratitis, cataracts, AMD<br>/ premature aging of the skin, wrinkles, age spots<br>/ Skin cancer, carcinomas, melanomas<br>/ Other / None / DNK / Refusal to answer<br><br>*Do not list answers*<br>*SEVERAL ANSWERS POSSIBLE*
Attitudes and beliefs	

For the following statements, indicate whether you strongly agree, tend to agree, neither agree nor disagree, tend to disagree or strongly disagree<br><br>a) I like sunbathing<br>b) I think I'm better looking when I've got a tan<br>c) Staying in the sun is good for my health<br>d) Staying in the sun will make my skin wrinkle sooner than expected<br>e) I feel better when I stay in the sun	Strongly agree / tend to agree / neither agree nor disagree / tend to disagree / strongly disagree / DNK / Refusal to answer
For the following statements, indicate whether you strongly agree, tend to agree, neither agree nor disagree, tend to disagree or strongly disagree<br>a) I can stay in the sun longer if I use sunscreen<br>b) If the weather is cloudy I have to protect myself from the sun<br>c) Sunburn prepares the skin for the sun<br>d) Sunburn during childhood has consequences when you're an adult.<br>e) The people who I care about encourage me to protect myself from the sun.<br>f) The people who I care about protect themselves from the sun.	Strongly agree / tend to agree / neither agree nor disagree / tend to disagree / strongly disagree / DNK / Refusal to answer
In your opinion, what is your risk of developing a sun-related health problem in the future?	Zero / Low / Medium / High / Very high / DNK / Refusal to answer
In your opinion, what is your risk in the future of prematurely getting marks like spots, wrinkles or sagging skin related to your exposure to the sun?	Zero / Low / Medium / High / Very high / DNK / Refusal to answer
During vacation, protecting myself from the sun is	Very difficult / difficult/ neither difficult nor easy/ easy / very easy / DNK /refusal to answer

Sun-exposure behaviors	
During your stay here, how many hours a day in total do you think you will spend in the sun on a sunny day, including visits, walks, sports activities, the beach, the swimming pool, etc. ?	I don't go in the sun / less than 30 mins per day<br>/ between [30 mins and 1 hour[per day / between [1 and 2 hours[per day<br>/ between [2h and 3h[per day / between [3h and 4h[per day<br>/ between [4h and 5h[per day / between [5h and 6h[per day<br>/ between [6h and 7h[per day / between [8h and 9h[per day<br>/ between [9h and 10h[per day / 10h per day or more<br>/ DNK / Refusal to answer
During your stay here, how many hours a day do you think you will spend in the sun between 12 p.m. and 4 p.m. on a sunny day?	I don't go in the sun between 12p.m. and 4p.m.<br>/ less than 30 min per day / between [30 min and 1 hour[per day<br>/ between [1h and 2h[per day / between [2h and 3h[per day<br>/ between [3h and 4h] per day / DNK / Refusal to answer
During your stay here, how many hours a day do you think you will sunbathe on a sunny day, that is to say sit or lie in the sun tanning?	I don't sunbathe with the intention of tanning<br>/ less than 30 mins per day<br>/ between [30 mins and 1 hour[per day / between [1 and 2 hours[per day<br>/ between [2h and 3h[per day / between [3h and 4h[per

	day<br>/ between [4h and 5h[per day / between [5h and 6h[per day<br>/ between [6h and 7h[per day / between [8h and 9h[per day<br>/ between [9h and 10h[per day / 10h per day or more<br>/ DNK / Refusal to answer	
**<u>If you don't sunbathe with the intention of getting a tan</u>**<br>Last summer, did you sunbathe with the intention of getting a tan?	Yes/No/DNK/Refusal to answer	
**<u>If you sunbathe with the intention of getting a tan</u>**<br>In the future, do you intend to stop sunbathing with the intention of getting a tan?	Yes/No/DNK/Refusal to answer	
During your stay here, how many hours a day do you think you will spend at the beach or at the swimming pool on a sunny day?	I don't go to the beach or the swimming pool<br>/ less than 30 mins per day<br>/ between [30 mins and 1 hour[per day / between [1 and 2 hours[per day<br>/ between [2h and 3h[per day / between [3h and 4h[per day<br>/ between [4h and 5h[per day / between [5h and 6h[per day<br>/ between [6h and 7h[per day / between [8h and 9h[per day<br>/ between [9h and 10h[per day / 10h per day or more<br>/ DNK / Refusal to answer	
Protection behaviors		
To protect yourself from the sun during your stay, do you have here in the campsite:<br>a) a hat / cap<br>b) sunscreen<br>c) sunglasses<br>d) a parasol or tent or shade sail	Yes / No / DNK<br>Yes / No / DNK<br>Yes / No / DNK<br>Yes / No / DNK	
When it is sunny <u>during your stay here</u>, do you think you will use the following methods to protect yourself from the sun when you are outside for more than 15 minutes<br>a) staying in the shade or under a parasol<br>b) avoiding sunny hours between 12 p.m. and 4 p.m.<br>c) putting on sunscreen every 2 hours<br>d) wearing sunglasses<br>e) wearing a hat or cap<br>f) wearing a t-shirt that covers the shoulders	Always/ Often / Sometimes / Rarely / Never / DNK / Refusal to answer	
**<u>If you always stay in the shade, or if you always or often use all the other ways to protect yourself (sunscreen, hat, glasses, t-shirt, avoiding risky times of the day),</u>**<br>Did you protect yourself from the sun like this last summer?	Yes/No/DNK/Refusal to answer	
**<u>If you don't always stay in the shade or if you don't always use all the other ways to protect yourself (sunscreen, hat, glasses, t-shirt, avoiding risky times of the day),</u>**<br>Have you thought about protecting yourself	Yes/No/DNK/Refusal to answer	**<u>If answer is "yes"</u>,**<br>What means of protection might you consider using more regularly in the future?

more regularly from the sun, or thought about using new means of protection in the future?		a) staying in the shade or under a parasol<br>b) avoiding sunny hours between 12 p.m. and 4 p.m.<br>c) putting on sunscreen every 2 hours<br>d) wearing sunglasses<br>e) wearing a hat or cap<br>f) wearing a t-shirt that covers the shoulders<br>Yes / No / DNK / Refusal to answer

Effects on health	
Have you been sunburned in the last 4 days?	Yes / No / DNK / Refusal to answer

PARENTS of young children	
Are you the parent of at least one child aged under 12 years old who is present during this stay?	Yes / No / DNK / Refusal to answer
**<u>If answer is yes</u>, we will now talk about the <u>youngest child</u> present during this stay**	
How old is this child?	
Is this child	A boy / a girl?
For the following statements, indicate for this child whether you strongly agree, tend to agree, neither agree nor disagree, tend to disagree or strongly disagree<br>a) My child is better looking when he/she is tanned<br>b) Staying in the sun is dangerous for my child<br>c) I have to protect myself from the sun to set an example for my child<br>d) During vacation, protecting my child from the sun is easy	Strongly agree/ tend to agree/ neither agree nor disagree/tend to disagree / strongly disagree /DNK / Refusal to answer
Do you have the following means of protection here in the campsite for this child:<br>a) a hat / cap<br>b) sunscreen<br>c) sunglasses<br>d) a parasol or a tent or shade sail	Yes / No / DNK
When it is sunny during your stay here, do you think you will use the following methods to protect this child from the sun when he/she is outside for more than 15 minutes?<br>a) ask him/her to stay in the shade or under an umbrella<br>b) avoid going in the sun during sunny hours between 12 p.m. and 4 p.m.<br>c) put sunscreen on him/her every 2 hours<br>d) put sunglasses on him/her<br>e) put a hat or cap on him/her<br>f) put a t-shirt on him/her covering his/her shoulders	Always/ Often / Sometimes / Rarely / Never / DNK / Refusal to answer

Comments :
