# Supplementary material for: Effect of an Appearance-Based vs. a Health-Based Sun-Protective Intervention on French Summer Tourists' Behaviors in a Cluster Randomized Crossover Trial: The PRISME Protocol
Source: Front Public Health. 2020 Nov 5;8:569857. doi: 10.3389/fpubh.2020.569857 (PMC7676153; doi:10.3389/fpubh.2020.569857)
Supplement: Supplementary Material 5 — T1 questionnaire. [file Data_Sheet_5.pdf]

Questionnaire T1

Identification of site	
Name of campsite	
Number of pitch	
Intervention group	0/1/2
Date and time of interview	
Interviewer's identification code	

Identification of participants	
Identification code of adult participant	
Adult's Family name – First name	
Identification code of child participant	
Child's Family name – First name	

Knowledge	
In your opinion, what are the ways to protect oneself from the sun?	Stay in the shade / Wear sunglasses / Wear a T-shirt / Wear a hat or cap / Put on sunscreen / Avoid the sunniest hours / Other / DNK / Refusal to answer<br><br>*Do not list answers*<br>*SEVERAL ANSWERS POSSIBLE*
In your opinion, in the summer in France, what are the most dangerous times of the day when it is better not to stay in the sun?	Before 9 a.m. / 9 a.m. to 10 a.m. / 10 a.m. to 11 a.m. / 11 a.m. to 12 p.m. / 12 p.m. to 1 p.m. / 1 p.m. to 2 p.m. / 2 p.m. to 3 p.m. / 3 p.m. to 4 p.m. / 4 p.m. to 5 p.m. / 5 p.m. to 6 p.m. / 6 p.m. to 7 p.m. / 7 p.m. to 8 p.m. / After 8 p.m. / None / DNK / Refusal to answer<br><br>*Do not list answers*<br>*SEVERAL ANSWERS POSSIBLE*
Without doing any specific physical activity or swimming, how often should a person put on sunscreen while in the sun?	Every 30 mins / Every hour / Every 2h / Every 3h / Every 4h / Once during the day / DNK / Refusal to answer
What do you think are the possible consequences on the body of intense exposure to the sun, both immediately after and in the longer term?	Sunburns, burns<br>/ Sunstroke, dehydration, heat stroke<br>/ Skin rashes, pimples, sun allergies<br>/ eye problems, conjunctivitis, eye inflammation, photokeratitis, cataracts, AMD<br>/ premature aging of the skin, wrinkles, age spots<br>/ Skin cancer, carcinomas, melanomas<br>/ Other / None / DNK / Refusal to answer<br><br>*Do not list answers*<br>*SEVERAL ANSWERS POSSIBLE*
**Attitudes and beliefs**	
For the following statements, indicate whether you strongly agree, tend to agree, neither agree nor disagree, tend to disagree or strongly disagree<br><br>a) I like sunbathing<br>b) I think I'm better looking when I've got a tan<br>c) Staying in the sun is good for my health	Strongly agree / tend to agree / neither agree nor disagree / tend to disagree / strongly disagree / DNK / Refusal to answer

d) Staying in the sun will make my skin wrinkle sooner than expected<br>e) I feel better when I stay in the sun	
For the following statements, indicate whether you strongly agree, tend to agree, neither agree nor disagree, tend to disagree or strongly disagree<br><br>a) I can stay in the sun longer if I use sunscreen<br>b) If the weather is cloudy I have to protect myself from the sun<br>c) Sunburn prepares the skin for the sun<br>d) Sunburn during childhood has consequences when you're an adult.<br>e) The people who I care about encourage me to protect myself from the sun.<br>f) The people who I care about protect themselves from the sun.	Strongly agree / tend to agree / neither agree nor disagree / tend to disagree / strongly disagree / DNK / Refusal to answer
In your opinion, what is your risk of developing a sun-related health problem in the future?	Zero / Low / Medium / High / Very high / DNK / Refusal to answer
In your opinion, what is your risk in the future of prematurely getting marks like spots, wrinkles or sagging skin related to your exposure to the sun?	Zero / Low / Medium / High / Very high / DNK / Refusal to answer
During vacation, protecting myself from the sun is	Very difficult / difficult/ neither difficult nor easy/ easy / very easy / DNK /refusal to answer

Sun-exposure behaviors	
Since our last meeting, how many hours a day in total have you spent in the sun on a sunny day, including visits, walks, sports activities, the beach, the swimming pool, etc. ?	There hasn't been one sunny day in the last three days /There has been sunny weather but I didn't go in the sun / less than 30 mins per day<br>/ between [30 mins and 1 hour[per day / between [1 and 2 hours[per day<br>/ between [2h and 3h[per day / between [3h and 4h[per day<br>/ between [4h and 5h[per day / between [5h and 6h[per day<br>/ between [6h and 7h[per day / between [8h and 9h[per day<br>/ between [9h and 10h[per day / 10h per day or more<br>/ DNK / Refusal to answer
Since our last meeting, how many hours a day have you spent in the sun between 12 p.m. and 4 p.m. on a sunny day?	There hasn't been one sunny day in the last three days /There has been sunny weather but I didn't go in the sun between 12 p.m. and 4 p.m.<br>/ less than 30 min per day / between [30 min and 1 hour[per day<br>/ between [1h and 2h[per day / between [2h and 3h[per day<br>/ between [3h and 4h] per day / DNK / Refusal to answer
Since our last meeting, how many hours a day have you sunbathed on a sunny day, that is to say sat or lay in the sun tanning?	There hasn't been one sunny day in the last three days /There has been sunny weather but I didn't sunbathe with the intention of tanning<br>/ less than 30 min per day / between [30 min and 1 hour[per day<br>/ between [1h and 2h[per day / between [2h and 3h[per day

<table>
<tr><td></td><td colspan="2">/ between [3h and 4h] per day / DNK / Refusal to answer</td></tr>
<tr><td><u>If you sunbathed with the intention of getting a tan</u>
In the future, do you intend to reduce sunbathing with the intention of getting a tan?</td><td colspan="2">Yes/No/DNK/Refusal to answer</td></tr>
<tr><td>Since our last meeting, how many hours a day have you spent at the beach or at the swimming pool on a sunny day?</td><td colspan="2">There hasn’t been one sunny day in the last three days
/There has been sunny weather but I didn’t go to the beach or the swimming pool
/ less than 30 mins per day
/ between [30 mins and 1 hour[per day / between [1 and 2 hours[per day
/ between [2h and 3h[per day / between [3h and 4h[per day
/ between [4h and 5h[per day / between [5h and 6h[per day
/ between [6h and 7h[per day / between [8h and 9h[per day
/ between [9h and 10h[per day / 10h per day or more
/ DNK / Refusal to answer</td></tr>
<tr><td>Protection behaviors</td><td colspan="2"></td></tr>
<tr><td>On sunny days since our last meeting, have you used the following methods to protect yourself from the sun when you’ve been outside for more than 15 minutes
a) staying in the shade or under a parasol
b) avoiding sunny hours between 12 p.m. and 4p.m.
c) putting on sunscreen every 2 hours
d) wearing sunglasses
e) wearing a hat or cap
f) wearing a t-shirt that covers the shoulders</td><td colspan="2">Always/ Often / Sometimes / Rarely / Never / DNK / Refusal to answer</td></tr>
<tr><td><u>If you haven’t always stayed in the shade or if you haven’t always used all the other ways to protect yourself (sunscreen, hat, glasses, t-shirt, avoiding risky times of the day),</u>
Have you thought about protecting yourself more regularly from the sun, or thought about using new means of protection in the future?</td><td>Yes/No/DNK/Refusal to answer</td><td><u>If answer is “yes”</u>,
What means of protection might you consider using more regularly in the future?

a) staying in the shade or under a parasol
b) avoiding sunny hours between 12 p.m. and 4 p.m.
c) putting on sunscreen every 2 hours
d) wearing sunglasses
e) wearing a hat or cap
f) wearing a t-shirt that covers the shoulders

Yes / No / DNK / Refusal to answer</td></tr>
<tr><td><u>If you haven’t always stayed in the shade,</u>

Why haven’t you always stayed in the shade during your vacation here?</td><td colspan="2">Cost of parasol
/ Difficulty finding shade
/ It stops me from getting a tan
/ It’s ineffective to protect somebody from the sun, it’s ineffective against sunburn
/ My activities (games, swimming, etc.) are all in the sun / I like, I prefer to be in the sun</td></tr>
</table>

	/ I didn't think about it, I forgot<br>/ I don't need to because I'm not sensitive to the sun (I have tanned or dark skin, I don't get sunburned)<br>/ I didn't need to because it wasn't sunny enough<br>/ Other / DNK / Refusal to answer<br>*Do not list answers*<br>*SEVERAL ANSWERS POSSIBLE*
	**<u>If other,</u>** specify
**<u>If you didn't always put on sunscreen,</u>**<br>When you didn't put on sunscreen, what were the reasons?	Cost / It stops me from getting a tan<br>/ Unpleasant on the skin / Stains clothes<br>/ Sticks to the sans / Contains chemicals harmful to my health<br>/ Ineffective for UV protection, ineffective against sunburn<br>/ I didn't think about it, I forgot<br>/ I don't need it because I'm not sensitive to the sun (I have tanned or dark skin, I don't get sunburned)<br>/ I didn't need it because it wasn't sunny enough<br>/ Other / DNK / Refusal to answer<br>*Do not list answers*<br>*SEVERAL ANSWERS POSSIBLE*
	**<u>If other,</u>** specify
**<u>If you didn't always wear sunglasses,</u>**<br>When you didn't wear sunglasses, what were the reasons?	Cost / Tan lines/<br>/ I don't need them because I don't have sensitive eyes / I lose them / I don't see well with them<br>/ I didn't think about them, I forgot<br>/ I didn't need them because it wasn't sunny enough<br>/ They don't suit me, I don't find them aesthetically appealing on me<br>/ Other / DNK / Refusal to answer<br>*Do not list answers*<br>*SEVERAL ANSWERS POSSIBLE*
	**<u>If other,</u>** specify
**<u>If you didn't always put a hat/cap on</u>**<br>When you didn't put a hat/cap on, what were the reasons?	Cost / Keeps me too warm / Stops me getting a tan / Tan lines / Ineffective for UV protection, ineffective against sunburn / Flies away with the wind/<br>/ I didn't think about it, I forgot<br>/ I don't need one because I'm not sensitive to the sun (I have tanned or dark skin, I don't get sunburned)<br>/ I didn't need one because it wasn't sunny enough<br>/ Other / DNK / Refusal to answer<br>*Do not list answers*<br>*SEVERAL ANSWERS POSSIBLE*
	**<u>If other,</u>** specify
**<u>If you didn't always wear a T-shirt,</u>**<br>When you didn't wear a T-shirt, what were the reasons?	Cost / Keeps me too warm / Stops me getting a tan / Tan lines / Ineffective for UV protection, ineffective against sunburn<br>/ I didn't think about it, I forgot<br>/ I don't need one because I'm not sensitive to the sun (I have tanned or dark skin, I don't get sunburned)<br>/ I didn't need one because it wasn't sunny enough<br>/ Other / DNK / Refusal to answer<br>*Do not list answers*<br>*SEVERAL ANSWERS POSSIBLE*

	**<u>If other,</u>** specify
**<u>If you did use sunscreen,</u>**<br>What was the sun protection factor of the sunscreen you used during your stay?<br>a) on your face<br>b) on your body	I didn't use sunscreen on my face/body / SPF 1-14 / SPF 15-29 / SPF 30-49 / 50-50 /DNK / Refusal to answer

Effects on health	
Have you had sunburn since our last meeting?	Yes/No/DNK/Refusal to answer
**<u>If answer is "yes",</u>**<br>Did these sunburn lead to :<br>a) pain<br>b) peeling skin<br>c) blisters<br>d) difficulty sleeping	None at all/ Slightly/ Moderately/ Strongly /DNK / Refusal to answer
Have you had other sun-related problems since our last meeting?	No, none / problems with my eyes / heatstroke, sunstroke, dehydration / Pimples, sun allergies / Other / DNK / Refusal to answer
**<u>If "other",</u>** specify	
**<u>If you had sunburn or another health problem,</u>**<br><br>Because of these health problems, did you need to<br>a) consult a doctor?<br>b) go to the pharmacy?<br>c) take treatment?	Yes/No/DNK/Refusal to answer<br>If treatment was taken, specify which :

PARENTS of young children	
**<u>If you are the parent of a child under 12 years old,</u> we will now talk about the <u>youngest child</u> present during the stay**	
For the following statements, indicate for this child whether you strongly agree, tend to agree, neither agree nor disagree, tend to disagree or strongly disagree<br>a) My child is better looking when he/she is tanned<br>b) Staying in the sun is dangerous for my child<br>c) I have to protect myself from the sun to set an example for my child<br>d) During vacation, protecting my child from the sun is easy	Strongly agree/ tend to agree/ neither agree nor disagree/tend to disagree / strongly disagree /DNK / Refusal to answer
Since our last meeting, when it was sunny, did you use the following methods to protect this child from the sun when he/she was outside for more than 15 minutes?<br>a) ask him/her to stay in the shade or under an umbrella<br>b) avoid going in the sun during sunny hours between 12 p.m. and 4 p.m.<br>c) put sunscreen on him/her every 2 hours<br>d) put sunglasses on him/her<br>e) put a hat or cap on him/her<br>f) put a t-shirt on him/her covering his/her shoulders	Always/ Often / Sometimes / Rarely / Never / DNK / Refusal to answer
What was the sun protection factor of the sunscreen this child used during the stay?<br>a) on his/her face<br>b) on his/her body	He/she didn't use sunscreen on his/her face/body / SPF 1-14 / SPF 15-29 / SPF 30-49 / 50-50 /DNK / Refusal to answer

Since our last meeting, has this child had sunburn?	Yes/No/DNK/Refusal to answer

Perception of the intervention	
When we came here a few days ago, you participated in prevention activities with my colleague. I will now list these activities and you will tell me if they did - not at all, slightly, moderately or strongly - encourage you to modify your behavior in terms of the sun in the future.<br><br>a) (intervention group 1) My colleague's explanation of possible health problems linked to the sun<br>b) (intervention group 1) The results of your test to determine what type of sun-sensitivity profile your skin has<br>c) (intervention group 2) My colleague's explanation of the possible consequences of the sun on physical appearance<br>d) (intervention group 2) Seeing your UV photo<br>e) Protection recommendations given by my colleague<br>f) (intervention group 2) Discussions with my colleague using advertisements presenting tanned people<br>g) Reading the intervention materials provided	None at all/ Slightly/ Moderately/ Strongly /DNK / Refusal to answer

Comments :
