# Supplementary material for: Effect of an Appearance-Based vs. a Health-Based Sun-Protective Intervention on French Summer Tourists' Behaviors in a Cluster Randomized Crossover Trial: The PRISME Protocol
Source: Front Public Health. 2020 Nov 5;8:569857. doi: 10.3389/fpubh.2020.569857 (PMC7676153; doi:10.3389/fpubh.2020.569857)
Supplement: Supplementary Material 6 — Information letter for adults. [file Data_Sheet_6.pdf]

Santé publique France

Dossier suivi par :
Direction des régions
Cellule Occitanie

Cécile Durand
Téléphone : 05 34 30 25 23
Courriel : cecile.durand@santepubliquefrance.fr

Références : DIRE 2019-E051
N° chrono DG : DiRE-19-D-0274

Saint-Maurice, le 20 JUIN 2019

LA LETTRE D'INFORMATION (adultes majeurs)
Etude PRISME : PRévention et Impact de l'exposition Solaire sur le littoral MEditerranéen

Madame, Monsieur,

Santé publique France est un établissement public chargé par la loi de protéger efficacement la santé des populations. Ses missions s'articulent autour de la surveillance de l'état de santé des français, la promotion de la santé et la réduction des risques sanitaires.

Dans le cadre de ses missions, Santé publique France met en œuvre l'étude PRISME sur le littoral méditerranéen d'Occitanie en 2019-2020, avec la contribution de l'agence régionale de santé (ARS) Occitanie, et la collaboration d'Epidaure (département prévention de l'institut du cancer de Montpellier) et d'IPSOS (prestataire d'enquête).

Cette étude vise à :

- décrire les connaissances, attitudes, comportements et effets sanitaires liés aux expositions au soleil des touristes lors de leur séjour estival,
- déterminer les facteurs qui influencent les comportements de protection solaire durant le séjour,
- comparer les connaissances, attitudes, comportements de protection solaire et effets sanitaires des touristes dans différents groupes ciblés par des interventions de prévention.

Vous êtes sollicité pour participer à cette étude car votre emplacement a été tiré au sort. Vous êtes entièrement libres de participer ou non.
Votre participation à cette étude contribuera à améliorer la connaissance des comportements des touristes français face au risque solaire et à orienter les stratégies de prévention pour réduire les comportements à risque et ainsi diminuer les impacts sanitaires des expositions solaires estivales.

En pratique, l'étude consiste à interroger au sein de 8 campings du littoral jusqu'à 2 personnes par emplacement de camping sélectionné aléatoirement :

- Une personne âgée de 18 à 55 ans,
- Un adolescent âgé de 12 à 17 ans.

Santé publique France

L'enquête se déroule sur deux étés consécutifs (2019 et 2020). Ainsi, votre participation à l'étude implique :

- de répondre à des **questionnaires** sur vos caractéristiques individuelles, vos connaissances, opinions et comportements vis-à-vis de l'exposition au soleil et de la protection solaire :
  - ✓ deux questionnaires d'environ 15-20 minutes administrés en face à face lors du séjour (en début et fin de semaine) par un enquêteur soumis au secret professionnel,
  - ✓ un troisième questionnaire en ligne adressé par mail ultérieurement, à la fin de l'été 2020.

- la prise de **mesures de la couleur de votre peau** lors du séjour par un enquêteur à l'aide d'un appareil de mesure totalement indolore apposé sur plusieurs zones de votre corps (visage, épaules, bras).

Par ailleurs, en fonction du groupe d'intervention dans lequel vous aurez été aléatoirement sélectionné et en complément de ce recueil de données, certains d'entre vous recevront un **entretien de prévention** d'environ 20 minutes sur les effets de l'exposition solaire et les moyens de prévention recommandés, délivré par un enquêteur spécialisé en prévention. Dans certains cas, une **photographie** en ultraviolet de votre visage sera prise par l'enquêteur puis vous sera remise. **Cet entretien et cette photographie ne seront donc pas proposés à tous les participants et ne constituent en aucun cas une prise en charge ou un outil de diagnostic médical.**

Ce dispositif met en œuvre un traitement de données à caractère personnel, fondé sur l'intérêt public, assurant la sécurité et la confidentialité de vos réponses et mesures et protégeant ainsi votre vie privée (Pour plus de précisions sur vos droits, votre participation libre à l'étude et la sécurité des données, cf. page 2-3).

Martial Mettendorff
Directeur général par intérim de Santé publique France

En savoir plus sur votre participation libre à l'étude, vos droits et la sécurité des données collectées

Pour ne pas alourdir le texte, nous nous conformons à la règle qui permet d'utiliser le masculin avec la valeur de neutre.

Votre participation libre à l'étude :

La participation à cette étude est volontaire. Il n'y aura pas de rémunération pour cette participation qui n'occasionne aucun frais pour vous.

Information préalable :

En amont de votre participation, vous recevez de la part de l'enquêteur une information sur les finalités et le déroulé de l'étude, ainsi que sur vos droits.

Acceptation :

Puis l'enquêteur sera chargé de recueillir votre accord oral à participer à cette étude.

Que deviennent vos informations personnelles collectées ?

- ***Données de contact et photographies UV :***

Votre identité (nom, prénom) et vos coordonnées (mail et téléphonique) sont recueillies uniquement afin de pouvoir prendre contact avec vous pour le déroulé de l'étude pendant et après votre séjour. Elles permettront de vous adresser deux mails d'informations, le dernier questionnaire en septembre 2020 et les résultats finaux.

Les photographies UV, le cas échéant, sont conservées afin de pouvoir vous être renvoyées ultérieurement lors de l'envoi des deux mails d'information.

Ces informations nominatives seront conservées dans une base de données sécurisée et distincte des données nécessaires aux analyses (données recueillies par questionnaires). Elles seront accessibles aux agents d'IPSOS en charge de la réalisation des interventions durant votre séjour puis aux agents de Santé publique France en charge de vous recontacter après votre séjour.

Ces informations nominatives seront détruites à l'issue de l'étude, soit au plus tard en février 2021. Seules seront conservées vos adresses mail (mais sans lien avec vos données) afin de vous envoyer les résultats de l'étude.

- ***Données recueillies par questionnaire / mesures de la couleur de peau :***

Ces données nécessaires à la réalisation des analyses sont conservées dans une base sécurisée distincte des données de contact. Les agents habilités de Santé publique France et d'IPSOS y auront accès. A l'issue du recueil, ces données seront conservées par Santé publique France à des fins d'analyse jusqu'en 2025, puis sous forme d'archives jusqu'en 2029.

- ***Sécurité et confidentialité des données***

Santé publique France et son prestataire IPSOS garantissent la sécurité et la confidentialité des données que vous nous confiez, de leur collecte à leur destruction. Les agents de Santé publique France et d'IPSOS sont soumis au secret professionnel.

- ***Résultats globaux de l'étude : garantie de l'anonymat***

En aucun cas les résultats globaux de l'étude ne permettront de vous identifier directement ou indirectement.

Quels sont vos droits ?

- ***Traitement des données***

Exercice de vos droits d'accès, de rectification, de suppression et de limitation

Conformément aux dispositions des articles 39 et 40 de la loi n°78-17 du 6 janvier 1978 relative à l'informatique, aux fichiers et aux libertés, vous pouvez exercer votre droit d'accès à ses données et éventuellement procéder à leur rectification et leur suppression, ou en limiter le traitement en vous adressant à Cécile Durand, investigateur principal de cette étude, à Santé publique France Occitanie (cecile.durand@santepubliquefrance.fr)

Santé publique France

Pour l'exercice de ces droits il vous sera demandé de justifier de votre identité. Vous pourrez exercer ces droits jusqu'à la suppression du fichier contenant les données nominatives détenus par Santé publique France (suppression prévue pour le 28/02/2021).

Exercice du droit d'opposition :
Conformément à l'article 38 de la loi n°78-17 du 6 janvier 1978 relative à l'informatique, aux fichiers et aux libertés, vous pouvez exercer à tout moment, et sans avoir à justifier de votre décision, votre droit d'opposition au traitement de vos données :

- Soit auprès de l'enquêteur IPSOS.
- Soit selon les mêmes modalités et conditions exposés, pour l'exercice des droits d'accès, de rectification, de suppression et de limitation.

Si vous décidez de vous opposer au traitement de vos données, vos informations nominatives seront détruites et vous ne pourrez notamment pas être recontacté par Santé publique France pour la participation à la dernière phase de l'étude.

Information et réclamations relatives au traitement de données
Pour plus d'information et renseignement sur le traitement de vos données, vous pouvez contacter le délégué à la protection des données (DPO) de Santé publique France : dpo@santepubliquefrance.fr.
En cas de réclamations vous pouvez saisir la Commission nationale informatique et libertés (CNIL). Nous vous conseillons cependant de prendre attache préalablement auprès de notre DPO qui est à votre disposition à cet effet.

➢ ***Accès aux résultats globaux de l'étude***

Sur demande, auprès de Santé publique France Occitanie, vous pourrez avoir un accès aux résultats globaux de l'étude.

Cadre réglementaire

Le traitement de données mis en œuvre est fondé sur l'intérêt public et a été autorisé par la CNIL (décision CNIL DR-2019-110 du 25 avril 2019 relative à la demande d'autorisation n°919075) sur le fondement du chapitre IX section 2 de la loi n°78-17 du 6 janvier 1978 relatif à l'informatique, aux fichiers et aux libertés. Ce traitement de données à caractère personnel n'a pas de caractère obligatoire.

Plus d'information sur l'étude :

Pour toutes autres questions relatives à l'étude, vous pouvez contacter Cécile Durand, investigateur principal de cette étude, à Santé publique France Occitanie (cecile.durand@santepubliquefrance.fr - tél. 05 34 30 25 23).
