# Supplementary material for: Effect of an Appearance-Based vs. a Health-Based Sun-Protective Intervention on French Summer Tourists' Behaviors in a Cluster Randomized Crossover Trial: The PRISME Protocol
Source: Front Public Health. 2020 Nov 5;8:569857. doi: 10.3389/fpubh.2020.569857 (PMC7676153; doi:10.3389/fpubh.2020.569857)
Supplement: Supplementary Material 7 — Information letter for minors. [file Data_Sheet_7.pdf]

Dossier suivi par :
Direction des régions
Cellule Occitanie

Cécile Durand
Téléphone : 05 34 30 25 23
Courriel : cecile.durand@santepubliquefrance.fr

Références : DIRE 2019-E052
N° chrono DG : DiRE-19-D-0275

Saint-Maurice, le 20 JUIN 2019

LA LETTRE D'INFORMATION (mineurs 12-17 ans)
Etude PRISME : PRévention et Impact de l'exposition Solaire sur le littoral MEditerranéen

Mademoiselle, Monsieur,

Santé publique France est un établissement public chargé par la loi de protéger la santé de la population française.

Santé publique France te propose de participer à l'étude PRISME qu'elle met en œuvre sur le littoral méditerranéen d'Occitanie en 2019-2020, avec la contribution de l'agence régionale de santé (ARS) Occitanie, et la collaboration d'Epidaure (département prévention de l'institut du cancer de Montpellier) et d'IPSOS (prestataire d'enquête).

Cette étude vise à :

- décrire les connaissances, attitudes, comportements et effets sur la santé liés aux expositions au soleil des touristes lors de leur séjour l'été,
- déterminer les éléments qui influencent les comportements de protection solaire durant le séjour,
- comparer les connaissances, attitudes, comportements de protection solaire et effets sur la santé des touristes dans différents groupes ciblés par des interventions de prévention.

Tu es sollicité pour participer à cette étude car ton emplacement a été tiré au sort. Tu es entièrement libre de participer ou non.
En participant à cette étude, tu contribueras à mieux connaitre les comportements des touristes français face au soleil, à identifier les messages de prévention efficaces pour améliorer les comportements de protection au soleil et ainsi diminuer l'apparition des maladies liées à l'exposition au soleil.

En pratique, l'étude consiste à interroger au sein de 8 campings du littoral jusqu'à 2 personnes par emplacement de camping sélectionné aléatoirement :

- Une personne âgée de 18 à 55 ans,
- Un adolescent âgé de 12 à 17 ans.

Santé publique France

L'enquête se déroule sur deux étés consécutifs (2019 et 2020). Ainsi, ta participation à l'étude implique :

- de répondre à des **questionnaires** sur tes caractéristiques individuelles (ton âge, sexe, département de résidence, les caractéristiques physiques notamment ton type de peau), tes connaissances, opinions et comportements vis-à-vis de l'exposition au soleil et de la protection solaire :
  - ✓ deux questionnaires d'environ 15-20 minutes administrés en face à face lors du séjour (en début et fin de semaine) par un enquêteur soumis au secret professionnel,
  - ✓ un troisième questionnaire en ligne adressé par mail ultérieurement, à la fin de l'été 2020.

- la prise de **mesures de la couleur de ta peau** lors du séjour par un enquêteur à l'aide d'un appareil de mesure totalement indolore apposé sur plusieurs zones de ton corps (visage, épaules, bras).

Par ailleurs, en fonction du groupe d'intervention dans lequel tu as été aléatoirement sélectionné et en complément de ce recueil de données, certains participants recevront un **entretien de prévention** d'environ 20 minutes sur les effets de l'exposition solaire et les moyens de prévention recommandés, délivré par un enquêteur spécialisé en prévention. Dans certains cas, une **photographie** en ultraviolet de ton visage sera prise par l'enquêteur puis te sera remise. **Cet entretien et cette photographie ne seront donc pas proposés à tous les participants et ne constituent en aucun cas une prise en charge ou un outil de diagnostic médical.**

Ce dispositif met en œuvre un traitement de données à caractère personnel, fondé sur l'intérêt public, assurant la sécurité et la confidentialité de tes réponses et mesures (colorimétrie et éventuellement photo UV), et protégeant ainsi ta vie privée. **Pour toi qui es mineur**, l'ensemble de tes droits (opposition, accès, rectification, limitation) sur tes données est exercé par tes représentants légaux (parents ou tuteurs légaux) (Pour plus de précisions sur tes droits, ta participation libre à l'étude et la sécurité des données, cf. page 2-3).

Martial Mettendorff
Directeur général par intérim de Santé publique France

En savoir plus sur ta participation libre à l'étude, tes droits et la sécurité des données collectées

Pour ne pas alourdir le texte, nous nous conformons à la règle qui permet d'utiliser le masculin avec la valeur de neutre.

Ta participation libre à l'étude :

La participation à cette étude est volontaire. Il n'y aura pas de rémunération pour cette participation qui n'occasionne aucun frais pour toi ou tes parents.

Information préalable :

Avant de ta participation, tu recevras de la part de l'enquêteur une information sur les finalités et le déroulé de l'étude, ainsi que sur tes droits. Cette information est également délivrée à un de tes représentants légaux (parents ou tuteurs légaux).

Acceptation :

Puis l'enquêteur sera chargé de recueillir ton accord oral à participer à cette étude. L'enquêteur devra également recueillir cet accord oral auprès d'un de tes représentants légaux (parents ou tuteurs légaux).

Que deviennent tes informations personnelles collectées ?

➢ ***Données de contact et photographies UV :***

Ton identité (nom, prénom) et tes coordonnées (mail et téléphone personnels ou ceux de tes parents si tu n'as pas de coordonnées personnels) sont recueillies uniquement afin de pouvoir prendre contact avec toi pour le déroulé de l'étude pendant et après ton séjour. Elles permettront de t'adresser deux mails d'informations, le dernier questionnaire en septembre 2020 et les résultats finaux.

Les photographies UV, le cas échéant, sont conservées afin de pouvoir t'être renvoyées ultérieurement lors de l'envoi des deux mails d'information.

Ces informations nominatives seront conservées dans une base de données sécurisée et distincte des données nécessaires aux analyses (données recueillies par questionnaires). Elles seront accessibles aux agents d'IPSOS en charge de la réalisation des interventions durant ton séjour puis aux agents de Santé publique France en charge de te recontacter après ton séjour.

Ces informations nominatives seront détruites à l'issue de l'étude, soit au plus tard en février 2021. Seules seront conservées ton adresse mail ou celle de tes parents (mais sans lien avec tes données) afin de t'envoyer les résultats de l'étude.

➢ ***Données recueillies par questionnaire / mesures de la couleur de peau :***

Ces données nécessaires à la réalisation des analyses sont conservées dans une base sécurisée distincte des données de contact. Les agents habilités de Santé publique France et d'IPSOS y auront accès. A l'issue du recueil, ces données seront conservées par Santé publique France à des fins d'analyse jusqu'en 2025, puis sous forme d'archives jusqu'en 2029.

➢ ***Sécurité et confidentialité des données***

Santé publique France et son prestataire IPSOS garantissent la sécurité et la confidentialité des données que tu nous confies, de leur collecte à leur destruction. Les agents de Santé publique France et d'IPSOS sont soumis au secret professionnel.

➢ ***Résultats globaux de l'étude : garantie de l'anonymat***

En aucun cas les résultats globaux de l'étude ne permettront de t'identifier directement ou indirectement.

Quels sont tes droits ?

➢ ***Traitement des données***

Exercice de tes droits d'accès, de rectification, de suppression et de limitation

Conformément aux dispositions des articles 39 et 40 de la loi n°78-17 du 6 janvier 1978 relative à l'informatique, aux fichiers et aux libertés, tes représentants légaux (parents ou tuteurs légaux) peuvent exercer un droit d'accès à ses données et éventuellement procéder à leur rectification et leur suppression, ou en limiter le traitement en s'adressant à Cécile Durand, investigateur principal de cette étude, à Santé publique France Occitanie (cecile.durand@santepubliquefrance.fr)

Dans le cadre de cet exercice, tes représentants légaux peuvent donc demander la communication de tes données.
Pour l'exercice de ces droits il sera demandé à tes représentants légaux de justifier de leur identité et de leur lien de parenté. Tes représentants légaux pourront exercer ces droits jusqu'à la suppression du fichier contenant les données nominatives détenus par Santé publique France (suppression prévue pour le 28/02/2021).

Exercice du droit d'opposition :
Conformément à l'article 38 de la loi n°78-17 du 6 janvier 1978 relative à l'informatique, aux fichiers et aux libertés, tes représentants légaux peuvent exercer à tout moment, et sans avoir à justifier de leur décision, un droit d'opposition au traitement de tes données :

- Soit auprès de l'enquêteur IPSOS.
- Soit selon les mêmes modalités et conditions exposés pour l'exercice des droits d'accès, de rectification, de suppression et de limitation.

Si tes représentants légaux décident de s'opposer au traitement de tes données, tes informations nominatives seront détruites et tu ne pourras notamment pas être recontacté par Santé publique France pour la participation à la dernière phase de l'étude.

Information et réclamations relatives au traitement de données
Pour plus d'information et renseignement sur le traitement de tes données, toi et tes représentants légaux pouvez contacter le délégué à la protection des données (DPO) de Santé publique France : dpo@santepubliquefrance.fr.

En cas de réclamations toi et tes représentants légaux pouvez saisir la Commission nationale informatique et libertés (CNIL). Nous vous conseillons cependant de prendre attache préalablement auprès de notre DPO qui est à votre disposition à cet effet.

➢ ***Accès aux résultats globaux de l'étude***
Sur demande, auprès de Santé publique France Occitanie, toi et tes représentants légaux pourrez avoir un accès aux résultats globaux de l'étude.

Cadre réglementaire

Le traitement de données mis en œuvre est fondé sur l'intérêt public et a été autorisé par la CNIL (décision CNIL DR-2019-110 du 25 avril 2019 relative à la demande d'autorisation n°919075) sur le fondement du chapitre IX section 2 de la loi n°78-17 du 6 janvier 1978 relatif à l'informatique, aux fichiers et aux libertés. Ce traitement de données à caractère personnel n'a pas de caractère obligatoire.

Plus d'information sur l'étude :

Pour toutes autres questions relatives à l'étude, toi et tes représentants légaux pouvez contacter Cécile Durand, investigateur principal de cette étude, à Santé publique France Occitanie (cecile.durand@santepubliquefrance.fr - tél. 05 34 30 25 23).
